# Walking enhances peripheral visual processing in humans

**DOI:** 10.1371/journal.pbio.3000511

**Published:** 2019-10-11

**Authors:** Liyu Cao, Barbara Händel

**Affiliations:** Department of Psychology, University of Würzburg, Würzburg, Germany; McGill University, CANADA

## Abstract

Cognitive processes are almost exclusively investigated under highly controlled settings during which voluntary body movements are suppressed. However, recent animal work suggests differences in sensory processing between movement states by showing drastically changed neural responses in early visual areas between locomotion and stillness. Does locomotion also modulate visual cortical activity in humans, and what are the perceptual consequences? Our study shows that walking increased the contrast-dependent influence of peripheral visual input on central visual input. This increase is prevalent in stimulus-locked electroencephalogram (EEG) responses (steady-state visual evoked potential [SSVEP]) alongside perceptual performance. Ongoing alpha oscillations (approximately 10 Hz) further positively correlated with the walking-induced changes of SSVEP amplitude, indicating the involvement of an altered inhibitory process during walking. The results predicted that walking leads to an increased processing of peripheral visual input. A second study indeed showed an increased contrast sensitivity for peripheral compared to central stimuli when subjects were walking. Our work shows complementary neurophysiological and behavioural evidence corroborating animal findings that walking leads to a change in early visual neuronal activity in humans. That neuronal modulation due to walking is indeed linked to specific perceptual changes extends the existing animal work.

## Abstract 2

Cognitive processes are almost exclusively investigated under highly controlled settings in which voluntary body movements are suppressed. New neurophysiological and behavioral data show that processing of peripheral visual input in humans is enhanced during walking.

## Introduction

Perception is not only a function of the stimulus but also very much influenced by internal factors such as arousal and attention. Recently, animal work has added an interesting dimension to this: the behavioural state, such as locomotion. In mice [1–9] as well as invertebrates [10–12], these studies have shown that neural responses from sensory areas drastically differ between locomotion and a still state beyond the influence of arousal [13,14]. Particularly, surround suppression has been reported to be decreased in mice during locomotion [7].

Unfortunately, our understanding of human visual processing in natural settings such as during walking is surprisingly limited. Behavioural studies [15] and some emerging electrophysiological work indicate a general link in humans between movement and cognition such as memory, attention [16–18], and perceptual processes [19–25]. Interesting work by Benjamin and colleagues suggests that the influence of locomotion on early sensory activity translates to humans. Contrary to the single-cell recording work in mice [7], their study provides evidence of an increased surround suppression effect during treadmill walking as compared to standing [26].

Despite the advancements in technology with regard to its mobility, it is still a great challenge to record physiological measures during free walking whilst controlling all input and output variables; we want to point out two important considerations. Firstly, eye movements are known to be highly influenced by walking movement [27,28] and at the same time greatly modulate sensory brain activity and perception [29]. If subjects are allowed to move freely (as compared to still states), the eye movements must be considered. Unfortunately, a stringent analysis of eye movements is often missing [26]. Secondly, most research has focused on the study of humans in stationary settings such as walking on a treadmill or stationary cycling [17,26,30,31], with very few exceptions [32–35]. The stationary setup in human studies is mostly used because the electrophysiological signal, as picked up with human EEG (electroencephalogram), can be corrupted by muscle activity and electrode movement [14]. However, reflecting on the core motivation of studying the interaction between locomotion and visual processing, natural walking is a crucial ingredient. Besides differences in mechanical and task-related demands between treadmill and natural walking, the most important and ecologically relevant difference is that during natural walking, the visual input from the surround is highly important, whereas for stationary treadmill walking or cycling, the visual input from the surround is largely negligible. This becomes obvious when considering navigational processes. During free walking, the visual input will greatly influence the motor output because walking direction, pattern, and speed will be largely based on visual information [36,37]. Therefore, studying the relationship between locomotion and visual processing during natural walking is more ecological than during treadmill walking, in which the circular relationship between sensory input and motor output is disrupted. In the current study, we combined the latest mobile EEG/EOG (electrooculogram) technology (for the assessment of brain activity and eye movements), mobile visual stimulation, and behavioural measurements to study human visual processing during free walking. Interestingly, peripheral visual input in particular is used for the assessment of the heading direction and navigation [38,39]. We therefore investigated visual processing in the periphery compared to central foveal input whilst additionally considering the influence of eye movements.

Assessing steady-state visual evoked potential (SSVEP), which is known to be a stimulus-locked signal originating from the early visual cortex [40], we could demonstrate in a first study that walking modulated how visual input from the periphery influences (suppresses) central foveal input in a contrast-dependent fashion. This modulation was paralleled by a behavioural effect in the concurrently probed target-detection performance. Moreover, we could show that the modulation was linked to alpha oscillations, indicating a downregulation of inhibitory processes during walking. Importantly, these findings led to the hypothesis that specifically peripheral visual processing is enhanced during walking, which was confirmed in a second (behavioural) study.

## Results

**Fig 1:**
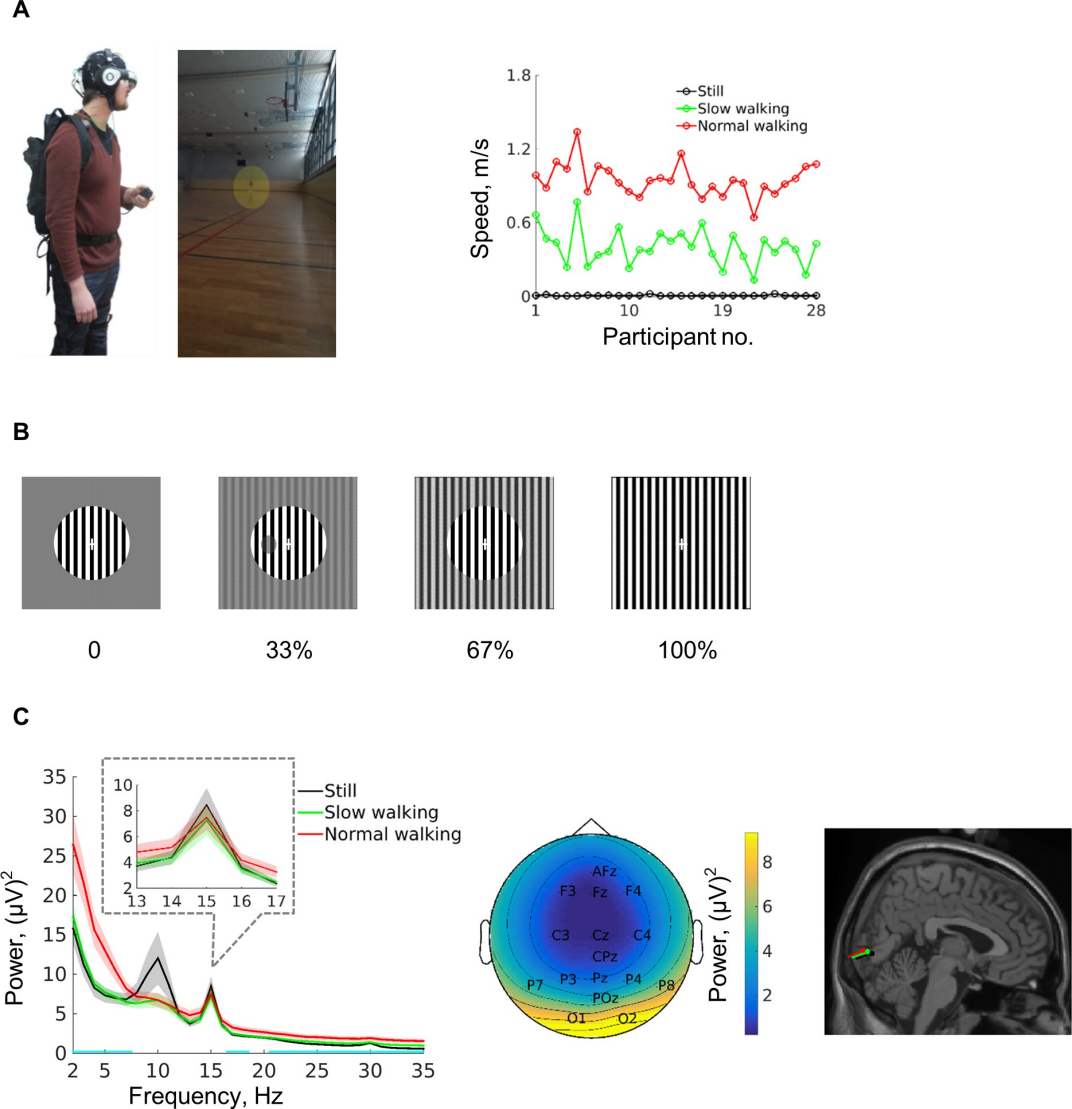
Experimental setup and SSVEP responses. (A) Participants carried all experimental equipment and walked freely in a 45 × 27 metre sports hall (the participant is marked with a yellow circle). Walking speed for the different walking conditions is shown on the right. (B) Illustration of the central flickering grating (visual angle: 6.1°) at four different levels of surround contrast (visual angle: 21.5°). An example contrast change (target) is shown in the second row. (C) Raw power spectrum of EEG responses in different walking conditions (left). Inset shows power responses around the SSVEP frequency of 15 Hz. Cyan lines mark the frequencies that showed power differences between walking conditions (*p* < 0.05, FDR-adjusted). Note that the power difference actually extends far beyond 35 Hz. Shading indicates ±1 standard error, *n* = 25 participants. Scalp topography of the average SSVEP response from all conditions is shown in the middle, and the source of the SSVEP response is estimated to be in the primary visual cortex in all conditions with dipole fitting (right). The original data are available from https://doi.org/10.6084/m9.figshare.9094742.v1. EEG, electroencephalogram; FDR, false discovery rate; SSVEP, steady-state visual evoked potential.

Participants were asked to stand still, walk slowly, or walk with normal speed on a self-chosen path whilst completing a perceptual task, which was presented via a head-mounted display (Fig 1A). Participants fixated on the centre of a circular grating flickering at 15 Hz (Fig 1B). The task was to detect a threshold-titrated contrast change (target) presented randomly in time and exact location but within the flickering central grating. A stable background grating presented at one of four different contrast levels between 0% and 100% surrounded the central grating.

Analysing the 2-second time window prior to target onset, an SSVEP response introduced by the central 15 Hz flickering grating was readily detected during standing as well as walking (as reported by previous studies testing the signal quality of mobile EEG setups [30,41]) and showed an occipital focus (Fig 1C). The source of the SSVEP was estimated to be in the primary visual cortex using a dipole fitting (dipole location in Montreal Neurological Institute [MNI] coordinates: [2.8, −84.7, −1.4] in standing still; [3.5, −88.8, 0.3] in slow walking; [2.9, −92.1, 1.3] in normal walking). Because walking led to an upshift of the EEG power spectrum, a relative SSVEP power was calculated by subtracting the average power of nearby frequencies to correct for walking associated noise. The relative SSVEP power, as well as the raw SSVEP power, was analysed further as a function of the two experimental manipulations, i.e., walking condition and surround contrast level.

### Walking modulates the surround-centre interaction as revealed by SSVEP response

**Fig 2:**
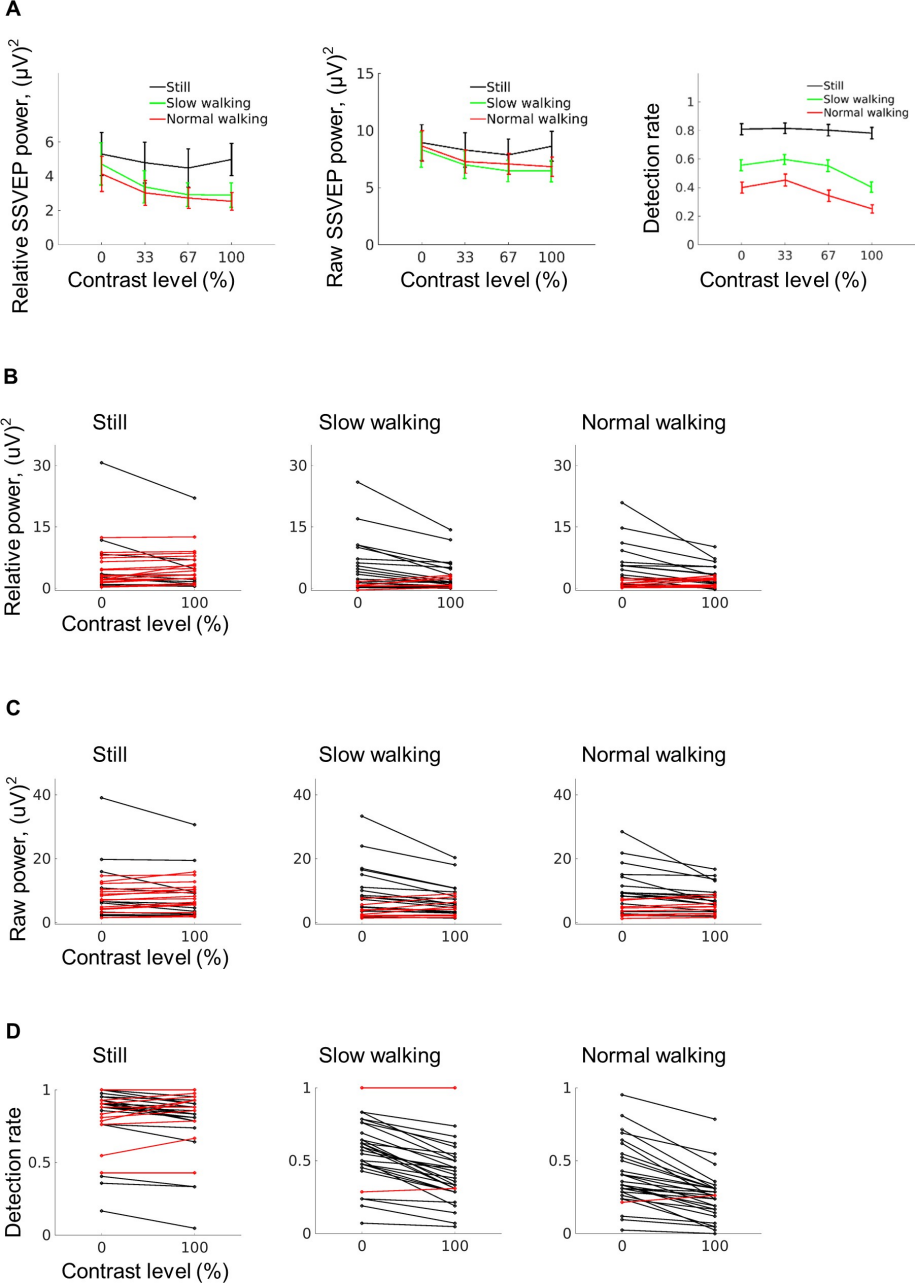
Influence of surround contrast depends on movement state. (A) SSVEP responses (left: referenced SSVEP power; middle: raw SSVEP power) and detection rates (right) significantly decreased with higher surround contrast levels during walking but not during standing still. Vertical lines indicate ±1 standard error, *n* = 25 (SSVEP); *n* = 30 (detection rate). Refer to S1 Table for break-down statistics. (B,C) Individual data from contrast level 0% and 100% for referenced SSVEP power and raw SSVEP power. Red lines indicate participants whose data patterns do not conform to suppression (detection rate at 100% contrast larger or equal to 0% contrast). (D) Same as B but showing the individual detection rate. The original data are available from https://doi.org/10.6084/m9.figshare.9094742.v1. SSVEP, steady-state visual evoked potential.

A within-subjects 2-factorial (3 × 4) ANOVA (F1: walking condition; F2: contrast level) revealed significant main effects (correction with Greenhouse–Geisser when the sphericity assumption was violated) (Fig 2A–2C). As expected [42], relative SSVEP power decreased with increasing contrast level (F[3, 72] = 5.56, *p* = 0.015; η_p_^2^ = 0.19; mean [SD]: 0%, 4.72 [5.75]; 33%, 3.73 [4.60]; 67%, 3.38 [3.78]; 100%, 3.47 [3.46]). Increasing walking speed was associated with significantly decreased relative SSVEP power (F[2, 48] = 8.04, *p* = 0.005; η_p_^2^ = 0.25; mean [SD]: still, 4.89 [5.54]; slow walking, 3.48 [4.42]; normal walking, 3.11 [3.43]). Most interestingly, there was a significant interaction between walking condition and contrast level (F[6, 144] = 3.03, *p* = 0.021; η_p_^2^ = 0.11). Post hoc analysis confirmed that relative SSVEP power was significantly decreased because of increased contrast level during walking but not during standing still, i.e., significant suppression from the periphery to centre was only observed whilst participants were walking (for detailed statistics, see S1 Table). A similar interaction effect was also found with the blink-controlled raw power at 15 Hz (F[6, 144] = 2.76, *p* = 0.032; η_p_^2^ = 0.10) (Fig 2A, middle). The interaction effect cannot be explained by either vision-nonspecific artefacts (because the effect was specific to the 15 Hz visually entrained signal; S1 Fig) or by head or eye movements (S2 and S3 Figs). Furthermore, a similar pattern was observed during target presentation (S1J Fig).

### Walking modulates the surround-centre interaction similarly in behavioural response

Our physiological data were paralleled by behavioural results (Fig 2A and 2D), which further reduces the likelihood of an artefact-based effect. The same within-subjects 2-factorial (3 × 4) ANOVA (F1: walking condition; F2: contrast level) revealed a significant interaction (F[6, 174] = 11.88, *p* < 0.001; η_p_^2^ = 0.29). In line with the neurophysiological finding, this suggests that perceptual suppression during walking is much stronger than during standing (for detailed statistics, see S1 Table). The detection rate further decreased with increasing walking speed (F[2, 58] = 108.46, *p* < 0.001; η_p_^2^ = 0.79; mean [SD]: still, 0.80 [0.21]; slow walking, 0.53 [0.20]; normal walking, 0.36 [0.20]) and contrast level (F[3, 87] = 66.05, *p* < 0.001; η_p_^2^ = 0.69; mean [SD]: 0%, 0.59 [0.19]; 33%, 0.62 [0.18]; 67%, 0.57 [0.19]; 100%, 0.48 [0.17]). Research suggests that walking cannot be fully automatic [43] and can impair spatial memory capacity and target-detection time [44]. The overall negative effect of walking on detection rate might be explained by dual task demands during walking. In this case, one might raise the concern of a nonlinear interaction between the detection rate and surround contrast, i.e., only weak perception might be subject to a suppression effect, whereas strong perception could be immune. In a follow-up analysis, we forced the detection rate for the 0-surround contrast to be similar between normal walking and standing still but still found a greater influence of surround contrast during walking (S4A Fig). This excludes the possibility that our finding is due to a nonlinear interaction between a changed perceptual threshold due to walking and the influence of surround contrast. No interaction effects between walking and surround contrast were found for false alarms or reaction time (S4B and S4C Fig), suggesting that a motor-based effect such as lowering the motor response threshold cannot explain the detection results.

### Positive interaction between SSVEP power and alpha power

Alpha power was greatly reduced during walking. A within-subjects one-way ANOVA confirmed a significant modulation of relative alpha power in individual peak alpha frequency by walking (F[2, 48] = 10.09, *p* = 0.003; η_p_^2^ = 0.30; mean [SD]: still, 1.68 [2.16]; slow walking, 0.80 [1.18]; normal walking, 0.42 [1.03]). Relative individual alpha power was lower under normal walking (*t*[24] = −3.34, *p* = 0.002; d_z_ = −0.67) and slow walking (*t*[24] = −2.87, *p* = 0.008; d_z_ = −0.57) conditions compared to the standing still condition. The normal walking condition also showed lower alpha power than the slow walking condition (*t*[24] = −3.46, *p* = 0.002; d_z_ = −0.69). The decrease of alpha power during walking, compared to standing, is a robust finding and is also statistically significant even if the raw alpha power in individual peak alpha frequency is compared (within-subjects one-way ANOVA with three walking conditions: F[2, 48] = 4.40, *p* = 0.045). We should bear in mind that the noise level may be different between different walking conditions, leading to a broadband change in power. Nevertheless, the alpha decrease during walking was significant with referenced power as well as the raw alpha power. This is important because depending on signal strength, as well as the amount of data available, increased noise may or may not affect the signal.

**Fig 3:**
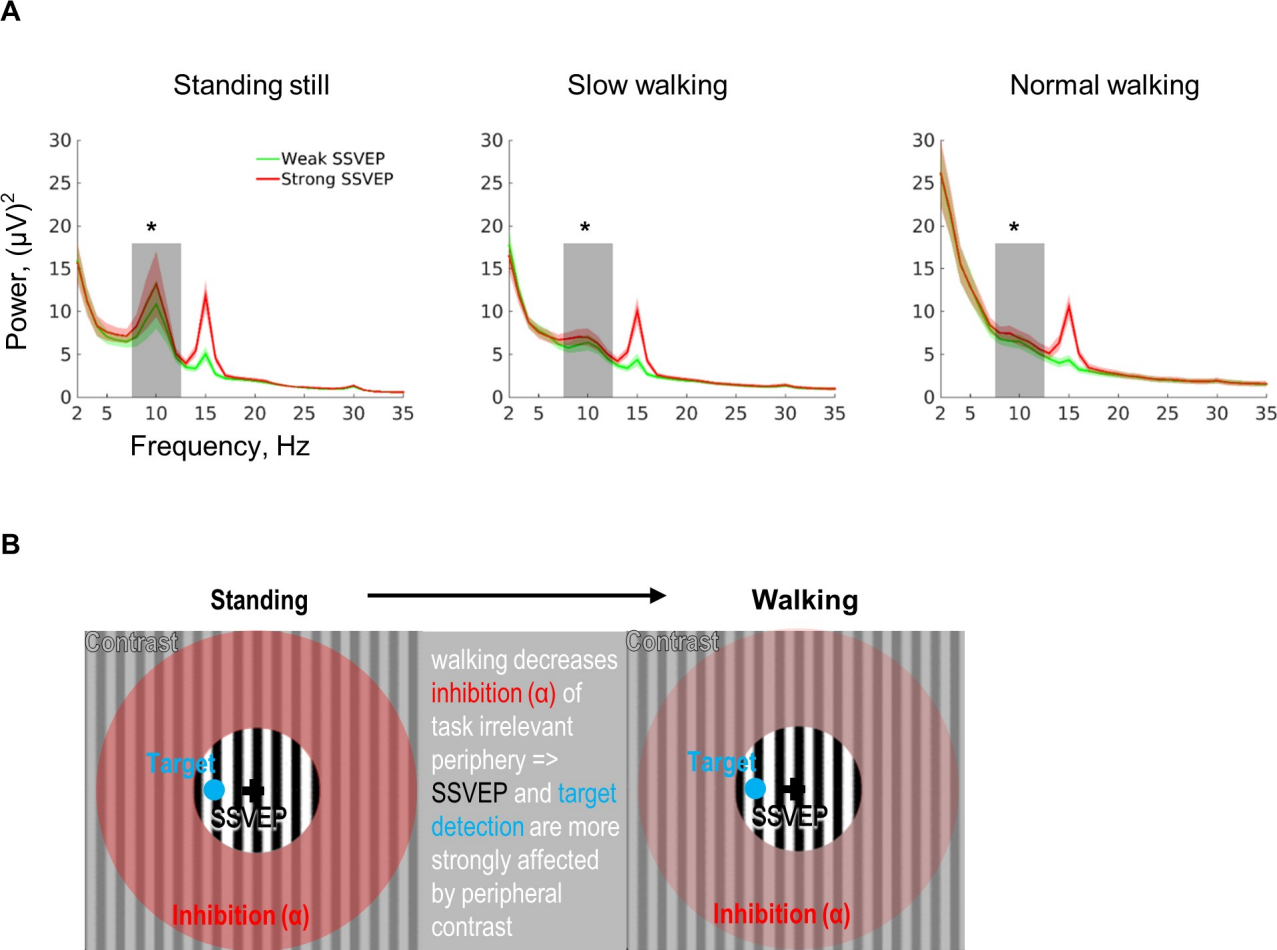
Interaction between SSVEP power and alpha power and the proposed mechanism of interaction. (A) In all three walking conditions, stronger SSVEP power was associated with stronger alpha power. No significant differences in other frequency bands were found. Shading indicates ±1 standard error. Grey bars indicate the alpha band (8–12 Hz), and asterisks indicate significant differences, *n* = 25 participants. (B) The diagram illustrates the suggested mechanistic influence of walking. Compared to standing still, walking leads to a decrease in inhibition indicated by alpha power (red area). This decreased inhibition gives a processing advantage to the peripheral visual field. In our specific stimulus, the peripheral visual field inhibits the central area (the strength depends on the background contrast). If the periphery is processed more strongly, the contrast of the background poses stronger influence on the central area. Consequently, SSVEP and behavioural detection rate (both exclusively probed and introduced in the central part of the visual field marked by the black circle) will decrease. The original data are available from https://doi.org/10.6084/m9.figshare.9094742.v1. SSVEP, steady-state visual evoked potential.

We next analysed the relationship between raw alpha and SSVEP power in each walking condition. Sorting trials based on their SSVEP power led to a significant difference in alpha power (but not in other frequency bands) in all three walking conditions (standing still: *t*[24] = −2.78, *p* = 0.010, d_z_ = −0.56; slow walking: *t*[24] = −3.87, *p* < 0.001, d_z_ = −0.77; normal walking: *t*[24] = −3.91, *p* < 0.001, d_z_ = −0.78). Strong alpha power was associated with strong SSVEP power (Fig 3A). A multiple linear regression analysis using alpha power and number of saccades and blinks as predictors and SSVEP power as response variable again showed that alpha power positively predicted SSVEP power (*t*[24] = 4.82, *p* < 0.001; mean [SD]: 0.07 [−0.02]), in addition to negative prediction effects from both saccades (*t*[24] = −2.86, *p* = 0.009; mean [SD]: −0.02 [0.03]) and blinks (*t*[24] = −5.48, *p* < 0.001; mean [SD]: −0.16 [0.15]).

### Walking leads to a processing advantage of peripheral stimuli

**Fig 4:**
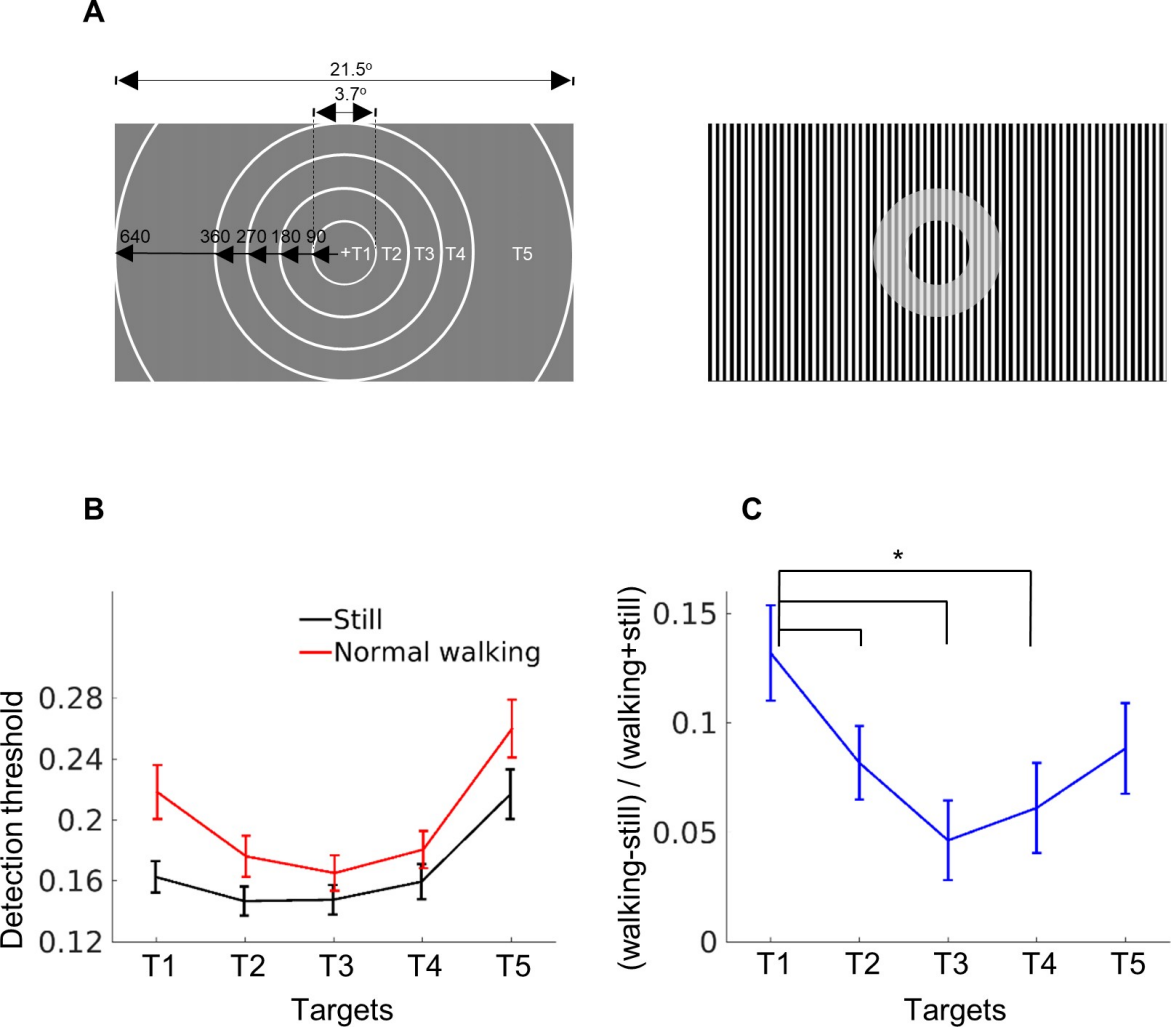
Walking leads to a processing advantage of peripheral stimuli. (A) Schematic illustration of the stimulus. Left, the size and position of all five targets (T1–T5) of contrast change are marked by circular white lines (not visible for the participant), corresponding to the ratio of real stimulus presentation. Stimulus size is given in pixel and visual angle. Right, an example of a T2 contrast change is shown. Note that the grating background was constantly at 100% contrast. (B) Detection threshold for all targets in both standing still and normal walking condition. (C) The relative detection threshold difference between normal walking and standing still for all targets. Each connected pair showed a significant difference (post hoc *t* test with FDR-adjusted *p*-values for multiple comparisons, *p* < 0.05). Vertical lines indicate ±1 standard error, *n* = 27 participants. The original data are available from https://doi.org/10.6084/m9.figshare.9094742.v1. FDR, false discovery rate.

Our finding that the suppressive effect of high contrast input from the periphery was strong during walking suggests that walking might in general raise the processing of peripheral input (by lowering inhibition as indicated by the observed alpha decrease) compared to central foveal input. In a follow-up experiment, we directly tested the hypothesis that peripheral stimuli receive enhanced processing during walking. Participants were asked to detect a contrast change presented at five different eccentricities (Fig 4A) during normal walking or standing still. Because dual task demands reduce the overall performance during walking, we did not necessarily expect to see an improvement in the detection rate in the periphery, but we did predict the difference in detection threshold between walking and standing to be smaller for the peripheral targets compared to the central target. Indeed, a significant interaction effect in a 2 (normal walking versus still) × 5 (target eccentricity) within-subjects ANOVA on detection threshold confirmed this prediction (F[4, 104] = 5.70, *p* = 0.002; η_p_^2^ = 0.18) (Fig 4B). The processing advantage for peripheral targets during walking as compared to standing still was found in all peripheral targets except target T5 (Fig 4C). Significant main effects were also present for walking condition (F[1, 26] = 27.95, *p* < 0.001; η_p_^2^ = 0.52; mean [SD]: still, 0.17 [0.06]; normal walking, 0.20 [0.07]) and target eccentricity (F[4, 104] = 41.50, *p* < 0.001; η_p_^2^ = 0.61; mean [SD]: T1, 0.19 [0.07]; T2, 0.16 [0.06]; T3, 0.16 [0.05]; T4, 0.17 [0.06]; T5, 0.24 [0.09]). The result considerably strengthens the hypothesis that peripheral stimuli, at least at certain eccentricities, receive enhanced processing during walking. The most peripheral target (T5) used here did not show a statistically significant processing advantage as compared to T1. This may suggest that there is a limit to the distance of peripheral visual field for receiving a processing advantage during walking.

## Discussion

With converging evidence from neurophysiological and behavioural measurements, we showed that the surround-centre interaction was much stronger in humans during walking compared to standing. Pretarget early visual SSVEP response (Fig 2A, left and middle), post-target SSVEP perturbation (S1J Fig), and behavioural target-detection rate (Fig 2A, right) were more strongly modulated by the surrounding contrast during walking. Neither eye movements nor a shift in signal-to-noise ratio as introduced by movement artefacts could account for these effects (S1–S3 Figs). The walking-induced change in surround-centre interaction could be caused by an altered processing of the peripheral input. A second behavioural study supported this idea of enhanced peripheral processing during walking by showing an increased contrast sensitivity in the periphery compared to the centre when walking. By uncovering a comodulation with alpha power, the current study further provides a tentative mechanism for the walking-induced effects, namely a modulation of inhibition as mediated by alpha oscillation.

### Walking, alpha power, and visual processing

Overall, alpha power significantly decreased during walking compared to standing still. A walking-induced alpha decrease has been noted previously [30,45,46], but a functional role was not experimentally tested. One influential account of alpha oscillations from neurocognitive studies proposes that alpha acts to functionally block out irrelevant information [47,48]. High alpha power is therefore associated with inhibiting sensory processing in a locally specific fashion [49]. Putting it differently, focused attention on a defined spatial region leads to an increase in alpha activity. We find that walking significantly decreased alpha power over occipital cortex, which, in turn, could suggest that attention is less focused on the foveal input by which the behavioural task is presented. Interestingly, both relative alpha power and relative SSVEP power decreased with increasing walking speed. This finding is consistent with the idea that whilst standing still, high alpha power may help inhibiting the influence from the periphery, thereby attenuating suppression (leading to high SSVEP power and decreasing the influence of surround contrast on detection rate and SSVEP). During walking, however, peripheral alpha power is decreased; thus, peripheral inhibition is compromised, and the surround contrast can exert its suppressive impact on the central grating (leading to low SSVEP power and a strong influence of surround contrast on detection rate and SSVEP) (see Fig 3B for a schematic illustration of the mechanism). The modulation of alpha power might be equivalent to one introduced by changes in attention. Interestingly, attention has been shown to have an attenuating effect on surround suppression in monkey V4 [50]. Unfortunately, our low number of electrodes does not permit us to differentiate between areas dealing with central versus peripheral vision by means of source localisation. However, the topography of alpha shows a focus of power over the occipital area overlapping with sites of strongest SSVEP power (S5A Fig). That walking in humans improves peripheral compared to central processing behaviourally could be viewed as an additional hint that walking triggers an attentional shift towards peripheral input. However, this does not necessarily mean that the net attentional processes are increased in the periphery; it might be just in comparison to central foveal input.

Biologically, it is rather plausible that walking enhances peripheral processing because input from the periphery holds important cues for locomotion and navigation [38,39]. Indeed, walking can influence how we perceive optic flow by decreasing the perceived speed of visual flow [20,23]. At the same time, we seem to be equally good at discriminating velocity increases in large accelerating flow fields independent of whether we stand, walk, or run [51]. In our experimental setup, limited optic flow was available from the upper and lower visual field during walking. Was it this extra visual input that affected visual processing, or are we rather dealing with a process that prepares the system for relevant input independent of the nature of this input? In the visual-target–detection performance of the SSVEP study, we did not find any evidence of an increased relevance for the upper or the lower visual target in terms of detection rate and electrophysiological response (see S4E and S4F Fig and discussion in S1 Text). Furthermore, in the second behavioural study, the relative improvement in detection performance during walking was most pronounced for target T3, which was not the closest target to the visible visual field. This argues against the notion that optic flow from the upper or lower visual field attracted more attention during walking. It also suggests that walking leads to a more general state-dependent change in processing the peripheral input.

But what specific process is affected by walking? Unfortunately, our experimental approach does not allow us to distinguish between a classical surround suppression effect and other suppression mechanisms such as overlay suppression [52]. This is discussed in detail in the section ‘Shortcomings of the current study’.

### The influence of walking speed

Motivated by animal work showing that the firing rate of a single visual cell can be modulated by locomotion speed [2], we included a slow walking and a normal walking condition in the SSVEP study. The suppression effect in both SSVEP response and behavioural detection rate does not appear to be different between slow walking and normal walking. It could be that the current measurement is not sensitive enough or suitable to detect subtle differences in visual processing between different speed conditions. Speaking in favour of a sensitivity issue, the alpha power is significantly modulated by the speed in the walking condition. The possible influence of walking speed on visual processing in humans needs further research.

### Brain oscillations in other frequencies during walking

Besides the described modulation of alpha oscillatory activity, the influence of walking on neural oscillations in other frequency bands is also interesting. The current study put its focus on the SSVEP response and alpha oscillations because the quality of the scalp-recorded EEG signal during walking still allows us to clearly show a visual origin of the signal. The sources of power modulation during walking in other frequency bands (e.g., theta and gamma) are much more difficult to assess especially because of movement-related noise; e.g., the topography of theta power showed an additional focus at the frontal electrode anterior frontal midline (AFz) besides an occipital focus (S5A Fig). During navigation, theta modulation can be found in frontal and temporal cortical subdural electrode contacts [53,54], and fronto-midline theta activity (picked up with surface EEG) has been shown to increase with walking [55,56]. This fronto-midline theta activity has been associated with working memory and spatial navigation [57,58]. Whilst we can be confident that the increase in theta power during normal walking (Fig 1C) is not introduced by the walking frequency (S5B Fig), an overall influence of movement-related artefacts cannot be excluded. Further studies, specifically targeting theta power, are therefore necessary before interpreting the theta modulation. Unfortunately, effects in the gamma range (Fig 1C), the most prominent oscillatory changes reported in the rodent literature associated with walking (e.g., [9]), also could not be fully dissociated from movement-related artefacts with the present study.

### Shortcomings of the current study

A surrounding stimulus usually has an inhibitory influence on a central stimulus, which is called the surround suppression effect [59,60]. Whilst surround suppression is classically described for centre and surround stimuli within the peripheral field, we used a stimulus that followed in large parts the study by Vanegas [42], who compared, amongst others, the effect of surround contrast on SSVEP power between peripheral and foveal locations. Despite the finding of a considerably weaker foveal suppression effect compared to the peripheral one, Vanegas and colleagues still showed a significant suppression even with a central foveal SSVEP target set to 100% contrast in behavioural and electrophysiological measurements [42], which is in line with previous psychophysical results [61]. Although using a very comparable stimulus, we could only find a significant influence of the surround contrast whilst subjects were walking but not during standing still. There is some quite interesting work discussing the specific role of central foveal stimuli compared to peripheral input with respect to surround suppression effects. Petrov and colleagues, who reported no surround suppression for foveal stimulation but strong suppressive effects of overlay suppression, argue for a clear distinction between foveal and peripheral suppressive influences [52]. We therefore suggest that our SSVEP effect might not be based on classical surround suppression but rather a combination with overlay suppression. This overlay effect might be introduced because of the absence of a blurring border between centre and surround stimuli, which serves the purpose of excluding the border contrast as a source of explanation [62]. However, importantly, additional control analysis did not indicate any influence from the border contrast itself (see S1K Fig), which means that the varying border contrast could not have been the source of response modulation. Furthermore, stimuli had a slightly increasing luminance with increasing contrast (approximately 5 lux between 0% and 100% contrast when holding a luminance meter before the lens of the head-mounted display in the eye’s position), which may introduce luminance as an additional confound. These issues may be relevant to the fact that no suppression effect was found in the standing condition (cf. [42]). Of course, the lack of suppression effect in the standing condition may also be due to the relatively large size of the central stimulus [62]. However, these issues are largely alleviated because the conclusion of the current study draws on the interaction effect between walking condition and surround contrast. Whatever problems the stimuli may have had, they are present equally in all conditions. Independent of the exact nature of the suppressive effect, our results show a significant modulation of suppressive influence due to walking.

### Control analysis on movement artefacts

Finally, we want to emphasise again the great importance of excluding the influence of movement artefacts on mobile EEG data. We conducted a large set of control analyses, which is included in detail in the S1 Text. Here, we want to shortly highlight the following points. 1) The interaction effect between walking condition and surround contrast on SSVEP response was specific for the entrained frequency (S1 Fig), which shows that the SSVEP signal (originating from visual cortex) specifically was modulated. 2) The modulation was unaffected by the walking-induced change of the broadband signal because the effect persisted with and without referencing to neighbouring frequencies (S1 Fig). 3) Our main finding, namely the interaction effect, is based on the power comparison between surround contrast conditions and not between walking conditions, which excludes any low-level influences of walking or stimulus confounds on our results. 4) We found no influence of head- or eye-related movements on our effect after carefully analysing head movements, blinks, and saccades with verified spatial sensitivity as small as 0.1° (S2 and S3 Figs). 5) The effect was not caused by a shift of gaze or attention towards the lower or upper visual field, where walking-relevant visual input such as optic flow is expected (S4 Fig).

To conclude, our study shows complementary neurophysiological and behavioural evidence that input from the peripheral visual field receives enhanced processing compared to the central foveal input during walking as compared to when standing still (with rigorous control analysis on eye movements). This reveals a critical and general difference in visual processing introduced by locomotion in humans. The current study further puts forward a cognitive and mechanistic explanation to the effect. Our results indicate that the walking-induced difference in processing is mediated by a modulation of inhibition indicated by changes in alpha oscillations. This might be equivalent to expanding the range of visual attention during walking. Our approach of free walking may be extended by applying recent exciting technological developments [63]. Overall, we propose that low-level processing of sensory information also crucially depends on the movement state of the subject and further emphasise the call for natural settings when investigating perceptual mechanisms and cognitive processes in general [28].

## Materials and methods

### Ethics statement

All participants gave written informed consent prior to the study and received monetary compensation after the study. The study was approved by the local ethics committee (Department of Psychology, University of Würzburg; reference number: GZEK 2015–01) and was conducted in accordance with the Declaration of Helsinki and the European data protection law (GDPR).

### Participants

30 healthy participants (20 females; mean age: 29.2; SD: 8.0) were recruited from a local participant pool for the SSVEP and walking study, and another 29 healthy participants (18 females; mean age: 26.4; SD: 6.9) were recruited for the follow-up behavioural study. The sample size was chosen so that reliable statistical results at the group level could be obtained because no prior studies were available for reference at the planning phase of the study.

### Stimuli and task

A circular grating (visual angle: 6.1°; spatial frequency: 2.5 cycles per degree; sine wave; 100% contrast) flickering at 15 Hz against a 0% contrast field was presented at the centre of the screen (refresh rate: 60 Hz; resolution: 1,280 × 720). The contrast level was defined as deviations from plain grey, which had a luminance level of 35 cd/m^2^ (0% contrast means plain grey and 100% contrast means alternations between plain white and plain black). The central grating was surrounded by a full-screen surround contrast (visual angle: 21.5°; spatial frequency: 2.5 cycles per degree; in phase with the central grating), which had one of the four following contrast levels: 0%, 33%, 67%, or 100%. The behavioural task was to detect a briefly presented target, which was a disk with a specific contrast appearing within the central grating (duration: 500 ms; visual angle: 1.2°; deviation from the screen centre: 2.0°). Participants were instructed to press a button as quickly as possible when a target was detected, independent of the location of the target. The target appeared randomly in one of four possible locations: above, below, or to the left or right of a central cross (visual angle: 0.3°), on which participants were required to keep fixation throughout the testing period. The contrast of the target was determined prior to the experiment using a 1 up, 4 down procedure [64], leading to a contrast for which the detection rate was theoretically at 84%. Participants completed the threshold test (duration approximately 5 minutes) with the surround contrast at 0% whilst sitting down. Stimuli were created and controlled with Psychtoolbox-3 in MATLAB (The MathWorks Inc., Natick, MA, USA).

Participants then completed the main task in three different walking conditions: standing still, walking slowly, or walking at a normal speed. There were seven testing blocks for each speed (21 blocks in total; random order). Before the start of each block, participants received instructions about the walking condition during the block. In each block, each of the four different surround contrast levels was presented for 35 seconds (random order). During each 35-second period, six targets were presented in the interval of 5–31.5 seconds with a stimulus-onset asynchrony randomly sampled between 3.5 and 6.5 seconds. After each block, participants were encouraged to take a break. A technical stop was made after blocks 7 and 14, during which the EEG electrode impedance was checked. After finishing the test, we also collected EEG data of free walking in a task-free setting during light or darkness and some questionnaires were completed (results will be reported elsewhere). Additionally, EEG data were recorded whilst subjects executed saccades between 0.1°–5° by following a saccade target presented on an external screen. The study was conducted in an activity hall (about 30 m × 50 metres; wooden floor) of the university gym. Because the lower rim of the visual field was unobstructed, participants could freely move around the large testing field using the information required for navigation [36] from the lower visual field.

For the follow-up behavioural test, the task was to detect a 500-ms contrast change on a background screen. The background consisted of a grating as previously described and was constantly kept at 100% contrast. Five eccentric areas (targets; note that now the target contrast change affects the entire area in between the different white lines shown in Fig 4A) were chosen in which the contrast change would occur. The smallest target was a circle at the screen centre with a diameter of 180 pixels (about 3.7°; screen resolution: 1,280 × 720). The other 4 targets were annulus-shaped, with the inner and outer diameter being (180, 360), (360, 540), (540, 720), and (720, 1,280), in pixels. The five targets covered the area from central visual field to peripheral visual field. During the test, contrast changes of target areas were presented randomly so no predictions about the location of change in the next trial could be formed, and all started with a contrast change of 0%. The stimulus-onset asynchrony was randomly sampled between 3,000 and 5,000 ms. If a button press was made within 1,000 ms from contrast change onset, it was considered as correct detection (hit). Otherwise, the contrast change was rated as missed. The amount of contrast change was controlled by a 1 up, 3 down procedure until 13 reversals were obtained. The step size was 6% before the fourth reversal and 3% after the fourth reversal. The contrast change levels from the last 10 reversal points were averaged to get an approximate 79% change detection threshold for each target. The detection threshold for each target was obtained in both the normal walking condition and the standing still condition. The two conditions alternated in sessions, each of which lasted about 5 minutes. The whole testing took about 40 minutes. In addition, participants were asked to always keep their fixation on a central cross during the testing. This part of testing was completed in an office hallway (10 m × 2.5 metres; flat uniform-grey floor) in the evening time with lights on. In the normal walking condition, participants walked between the two ends of the hallway; in the standing still condition, participants stood still in the middle part of the hallway. Only the behavioural responses were recorded for this test.

### Equipment

A Dell laptop (model: Latitude E7440) was used for running the experimental program and for data collection. During the experiment, the laptop was put into a rucksack, which was carried by participants to ensure full mobility during the testing. The screen of the laptop, which was used for stimulus presentation, was projected onto the participants’ eyes through a video headset (Glyph Founder’s edition; Avegant Corporation, Redwood, CA, USA). Prior to testing, the distance between the left and the right lens was adjusted by participants themselves to achieve binocular vision (verified by successful reading of small texts on the screen). Behavioural responses were collected using a handheld response button (model: K-RB1-4; The Black Box ToolKit Ltd, Sheffield, UK), which was connected to the laptop via USB. EEG data were collected using a Smarting mobile EEG system (mBrainTrain LLC, Belgrade, Serbia), which had 24 recording channels with a sampling rate of 500 Hz. We used six channels for EOG recording (for each eye: one below and one above the eye, one to the outer canthus), two channels for possible re-referencing (attached to both earlobes; eventually not used for the study), and the remaining 16 channels for EEG recording (see Fig 1C for EEG channel distribution). A common mode sense active electrode placed between Fz and Cz was used for online reference. The EEG signal amplifier and data transmitter are integrated into a little box (82 × 51 × 12 mm; 60 grams), which is attached to the back of the EEG cap. Data transmission is achieved via bluetooth. The EEG system also has a build-in gyroscope, which was used to measure head movements. Motion data (speed and acceleration; sampling rate: 120 Hz) were collected using a Perception Neuron system (Noitom Ltd, Beijing, China). Three functional motion sensors were attached to participants’ back and left and right ankles. The software Lab Streaming Layer (https://github.com/sccn/labstreaminglayer) was used for collecting and synchronising trigger output (indicating trial timing), behavioural responses, the EEG stream, and motion data. See Fig 1A for an illustration of the setup.

### Data analysis

Data analysis was performed with the Fieldtrip toolbox [65] and in-house scripts. Results of statistical analysis were reported following the guidelines of the American Psychological Association (APA). The within-subjects ANOVA was performed using SPSS-22 (IBM, Armonk, NY, USA) (Greenhouse–Geisser correction was performed when the sphericity assumption was violated). Throughout the manuscript, statistical cutoff was taken at the *p*-value of 0.05, and all *t* tests are two-tailed.

EEG data from five participants were incomplete because of data transmission error; therefore, only the remaining 25 full EEG datasets were included for the following analysis. For each participant, continuous EEG data were first cut into 35-second epochs, which covers the full duration of each surround contrast level (84 epochs per participant: 3 speeds × 7 blocks × 4 surround contrast levels). The data were then high-pass–filtered at 1 Hz (a windowed sinc finite-impulse–response filter with Kaiser window was used for all the filtering processes unless otherwise stated) and low-pass–filtered at 100 Hz. Noisy epochs (identified through visual inspection) were repaired through interpolation. 124 epochs in total (from six participants) were repaired. Amongst them, eight epochs (from three participants) were included in the SSVEP analysis (see below). An ICA approach to remove muscle-related noise was omitted because we did not find this approach to considerably improve data quality. Next, data were further segmented into 2-second snippets ending at the target onset time point. All the analyses below were based on these 2-second trials that are free from stimulus changes (i.e., target onset) and button press responses (trials with a button press in the 2.5-second window before the target onset were excluded). On average, 496.0 trials (SD: 11.0) remained for each participant (504 trials before rejection).

EEG power spectrum was obtained using the Welch’s method (1-second windowing with 50% overlap; ‘pwelch’ function in MATLAB). For each participant, three channels from the occipital area with highest mean power of all trials at 15 Hz were selected for analysing SSVEP and alpha power. A further trial exclusion was performed within each movement speed condition based on the high-frequency power using the median absolute deviation from median (MAD–median) rule: let p be the sum of the high-frequency power (20–99 Hz) of a single trial and P be the high-frequency power of all trials within a speed condition. If |p − median(P)| × 0.6745 > 2.24 × MAD–median, this trial is an outlier [66]. On average, 450.5 trials (SD: 24.7) remained after this step.

Source estimation of the SSVEP response was performed separately for each walking condition using the average power at 15 Hz from the 25 participants. Because no anatomical MRI was taken, we used the standard boundary element method volume-conduction model [67]. The location of a single dipole was estimated for each condition and was superimposed on a brain template (the Colin 27 brain, [68]). The residual variance of the dipole fitting in all conditions was very low (5.7%, 8.0%, and 6.9% in standing, slow walking, and normal walking conditions, respectively).

A within-subjects ANOVA was performed to compare the power of each frequency (between 2 and 35 Hz in steps of 1 Hz) amongst the three walking conditions (Fig 1B). *p*-Values from multiple comparisons were adjusted with FDR [69]. Conclusions are exclusively based on the statistical approach as described below. The SSVEP power at 15 Hz was then referenced to the mean power of nearby frequencies (13, 14, 16, and 17 Hz) through subtraction. The relative SSVEP power was averaged within each speed/surround contrast combination before being subjected to a 3 (walking condition) × 4 (contrast level) within-subjects ANOVA. To control for the influence of blinks (shown in Fig 2A, middle) and saccades (S2C Fig), trials with the same number of blinks or saccades were averaged before a grand average was taken. The relative alpha power was obtained similarly using power of frequencies 10, 11, and 12 Hz as reference.

To find the association between SSVEP power and alpha power, trials were grouped based on the median raw SSVEP power into weak SSVEP and strong SSVEP trials separately for each speed condition. For both slow and normal walking conditions, five trials with the lowest high-frequency power in the weak SSVEP group and five trials with the highest high-frequency power from the strong SSVEP group were excluded. This eliminated the group difference in the high-frequency band but did not change the statistical results comparing group differences in the other frequency bands. Possible power difference in the delta band (2–3 Hz), theta band (4–7 Hz), alpha band (8–12 Hz), and high-frequency band (20–99 Hz) was compared using within-subjects *t* tests across participants. Furthermore, a multiple linear regression analysis was performed for each participant, taking the SSVEP power of a single trial as the response variable and alpha power (from individual peak alpha frequency) and numbers of blinks and saccades as predictors. Both SSVEP power and alpha power were normalised within each testing block (i.e., fixed speed) to account for problems of power shifts between different speed conditions. Statistics was performed by making a group-level *t* test between the slope parameter and 0.

To get the target-evoked SSVEP perturbation, SSVEP amplitude was obtained through a Hilbert transform of the band-pass (14.5–15.5 Hz)–filtered data on each 35-second epoch (a windowed sinc finite-impulse–response filter with Hamming window). Then the target-evoked SSVEP perturbation was obtained through averaging trials aligned to the target onset. The amplitude of target-evoked SSVEP perturbation was calculated as the mean SSVEP amplitude between 200 and 600 ms after the target onset.

### Behavioural responses

A hit response was recorded if a button press was made within 1 second from the onset of the target. All other responses during the testing were regarded as false alarms. A detection rate, i.e., the number of hit responses divided by the number of targets, was calculated for each speed/surround contrast combination before being subjected to a 3 (walking condition) × 4 (contrast level) within-subjects ANOVA. Data from all 30 participants were included for analysis. False alarms and reaction time data (five participants were excluded based on the criterion that less than five responses were made in at least one condition) were analysed similarly. Note that arcsine-transformed detection-rate data and square-root–transformed data for false alarms resulted in similar statistical results. Therefore, no data transformation was performed for the reported statistics.

A target preference index was calculated for each participant in each walking condition and contrast level combination based on the detection rate of each target: target preference index = $abs(\sum_{i=1}^{4}\left(T-t_{i}))/(6*T\right)$, where *T* is the average detection rate of all four targets and *t*_*i*_ is the detection rate of each target (one participant was excluded because no targets were detected in at least one condition, which led to the denominator of the target preference index calculation being 0). This should give a preference index of 0 when detection rates for all the targets were the same and a preference index of 1 when detections were made only for one of the targets. The preference calculated in this way is sensitive to the number of trials being detected so that the preference index would be intrinsically larger when only very few targets are detected. Therefore, a simulation (1,000 times) was also run for each calculated preference index based on the assumption that the distribution of detected targets over locations are purely random. A *p*-value can then be obtained by calculating the percentage of the simulated preference index that is larger than the real preference index.

To test whether increased suppression during walking is simply due to the overall decrease of detection rate, we focused our analysis on the target (selected individually for each participant) with the lowest detection rate in the standing still condition and the target with the highest detection rate in the normal walking condition. We further excluded the first nine participants in the rank of detection-rate difference under 0 surround contrast between the standing still and normal walking conditions, after which a comparable detection rate under 0% surround contrast between standing still and normal walking was achieved for the remaining 21 participants. Comparisons of detection rate were then continued for the other 3 contrast levels.

For the follow-up behavioural test, the obtained detection thresholds were analysed with a 2 (normal walking versus still) × 5 (target size) within-subjects ANOVA. Two participants were excluded (incomplete datasets because of experimenter error/technical error), leaving a total of 27 participants in the final analysis. Because a significant interaction effect was found, a relative detection threshold difference was calculated for each grating contrast (target) to explore the interaction. The relative detection threshold difference as calculated as a ratio: (walking − still)/(walking + still). Within-subjects *t* tests were used to compare the detection threshold difference between different targets.

### Walking speed

The three-dimensional speed information from the motion sensor placed on the back of the subject was used to calculate the mean walking speed (Fig 1A). For calculating the power spectrum of walking speed data, we used the sum of walking speeds measured from both ankles (Welch’s method; S5B Fig). Data from two participants were missing because of technical errors during the recording.

### Blink detection

Blinks were detected from the vertical EOG component, i.e., the amplitude difference between the EOG channels above and below eyes. The vertical EOG component was high-pass–filtered at 0.2 Hz and low-pass–filtered at 20 Hz. A blink was detected if the vertical component crossed a threshold of 20 μV. Blinks with peak amplitude lower than 40 μV or amplitude SD smaller than 15 μV were excluded. Adjacent blink points within 100 ms were combined into one blink. Results of blink detection from both eyes were quite similar, and therefore, only results from the left eye were used.

### Saccade detection

Saccade detection was based on the so-called REOG signal, which is the difference between the mean of all six EOG channels and the Pz channel. REOG signal was band-pass–filtered between 20 and 90 Hz using a sixth-order Butterworth filter, and then a Hilbert transform was performed to obtain the amplitude envelop. All data points at which the amplitude value deviated from the mean by 2.5 SDs were considered saccade-related and were grouped into one saccade if they were less than 20 ms apart. This was done separately for each of the 21 recording blocks. This method was shown to be able to detect even small saccades very reliably [70].

Each saccade, as detected by means of the amplitude SD, defined a time window. An 80 ms filtered REOG signal was extracted centring at the lowest point of the filtered REOG signal in this time window. The 80 ms signal was then normalised by dividing the amplitude value at each time point by the session mean amplitude of the Hilbert-transformed REOG signal. The session mean amplitude of the Hilbert-transformed REOG signal was calculated separately for each of the 21 sessions during SSVEP testing. This referencing procedure was introduced to correct for any general power difference between different conditions. Averaging the 80 ms signal over detected saccades resulted in a clear saccade-related spike potential for each walking conditions (S2D Fig).

The average saccade size was estimated for each walking condition based on the saccadic spike potential obtained in the controlled saccade testing session that followed the walking experiment. In separate sessions, participants made 20 saccades in five different sizes (0.1°, 0.2°, 0.5°, 1.0°, and 5.0°) and four different directions (horizontal, vertical, and two diagonal directions) by following dots presented on a computer screen (20 × 5 × 4 saccades in total). Saccadic spike potentials were obtained for each saccade size using the procedure as described above. The size of controlled saccades and the group average amplitude of saccadic spike potential (using the amplitude value at time 0) were fitted with a second-order polynomial. Group average saccade sizes in different walking conditions were then estimated based on the fitting function.

### Strong head movement detection

Strong head movements were detected based on the gyroscope data. Time points with the rotational velocity deviating from the mean deviations (calculated within each testing block) by 2.5 SDs are regarded as time points of strong head movement. Strong head movement time points within a 2-second window were assigned to one head movement event.

## Supporting information

S1 Fig Control analysis of SSVEP. (A) Referenced SSVEP power without controlling for eye movements also showed a significant interaction between walking condition and surround contrast. (B) *p*-Values for the interaction effect between walking condition and contrast level for signals from 3 to 30 Hz (step size 1 Hz) processed in the same way as for the 15 Hz relative SSVEP signal. All frequencies had *p*-values above 0.05 except the signals of 15 and 17 Hz, the latter of which took contribution from 15 Hz signal (signals from each frequency were referenced to the mean of four nearby frequencies). (C) The relative power at 17 Hz (referenced to the average power of 15, 16, 18, and 19 Hz) also showed a significant interaction effect between walking condition and contrast level. This effect is likely driven by the SSVEP signal at 15 Hz. Note the negative sign of the relative power and the positive influence of surround contrast. (D) *p*-Values for the interaction effect between walking condition and contrast level for the raw power from 10 to 20 Hz (step size 1 Hz). All frequencies had *p*-values above 0.05, except the signal of 15 Hz. (E–I) Raw power shown in each walking condition and surround contrast combination for 13–17 Hz. (J) Amplitudes for target-evoked SSVEP perturbation (averaged between hit and miss trials; sign reversed; amplitude taken as the lowest amplitude point in a post-target time window of [0.2, 1] second). Note that another four participants were excluded (21 participants remained) because no hit or miss trials could be found in at least one condition for them. (K) EEG signal aligned to the onset of the last central contrast before the onset of the behavioural target, i.e., time 0 is the onset time of a central contrast. It is clear that the phase of EEG signal is aligned between all levels of surround contrast in all walking conditions. Border contrast elicited response would lead to a 180° phase difference between the 0% contrast and the 100% contrast condition. Blue colour: 0% contrast; red: 33% contrast; yellow: 67% contrast; purple: 100% contrast. Vertical lines indicate ±1 standard error. EEG, electroencephalogram; SSVEP, steady-state visual evoked potential.
(TIF)

S2 Fig Control analysis of saccades. (A) Trials were grouped based on the number of saccades detected within each trial (97.4% of all trials from 25 participants included. Trials showing more than eight saccades were not considered). SSVEP relative power decreased with increased number of saccades per trial. (B) Average number of saccades in each trial for each walking condition/contrast level combination. Only main effects of walking condition (F[2, 48] = 22.95, *p* < 0.001; η_p_^2^ = 0.49) and contrast level (F[3, 72] = 3.78, *p* = 0.018; η_p_^2^ = 0.14) were significant. *n* = 25 participants. (C) SSVEP in walking condition/contrast level combination with the potential influence from saccades controlled. (D) (Left) The saccadic spike potential (the U-shaped waveform) averaged across 25 participants for controlled saccades to visually presented targets (saccade amplitude: 0.1°, 0.2°, 0.5°, 1.0°, and 5.0°) and for saccades in each walking condition. The amplitude value at time 0 was used for the fitting shown on the right. (Right) The group average saccade size in each walking condition was estimated based on the amplitude at time point 0 of the controlled saccades with known sizes. The blue dots represent the controlled saccades, and the blue line is the fitting curve. Vertical lines indicate ±1 standard error. SSVEP, steady-state visual evoked potential.
(TIF)

S3 Fig Control analysis of blinks and head movements. (A) Trials were grouped based on the number of blinks detected within each trial (95.2% of all trials from 25 participants). SSVEP relative power was lower in no-blink trials than in trials with one blink. (B) Average number of blinks in each trial for each walking condition/contrast level combination. Only the main effect of walking condition (F[2, 48] = 4.21, *p* = 0.038; η_p_^2^ = 0.15) was significant. *n* = 25 participants. (C) SSVEP in walking condition/contrast level combination with the influence from blinks controlled. This is the same figure as Fig 2A (left) in the main text. (D) Trials were grouped based on the number of strong head movements detected within each trial (all trials from 25 participants). The SSVEP relative power did not change with number of strong head movements (*t*[24] = −0.45, *p* = 0.659). (E) There was no significant interaction effect for strong head movements between walking condition and surround contrast (F[6, 144] = 0.95, *p* = 0.449). The main effects of walking condition (F[2, 48] = 1.68, *p* = 0.199) and contrast level (F[3, 72] = 0.37, *p* = 0.766) were also not significant. Vertical lines indicate ±1 standard error. SSVEP, steady-state visual evoked potential.
(TIF)

S4 Fig Control analysis of behavioural target-related performance. (A) When the detection rate was forced to be comparable at 0% surround contrast (*t*[20] = 0.15, *p* = 0.882), detectable differences at 67% (*t*[20] = 2.54, *p* = 0.020; d_z_ = 0.55) and 100% (*t*[20] = 3.57, *p* = 0.002; d_z_ = 0.78) surround contrast can still be found between the still and normal walking conditions. A significant interaction between surround contrast and walking speed was also present (F[3, 60] = 3.19, *p* = 0.040; η_p_^2^ = 0.14). *n* = 21 participants. (B) Number of false alarms (calculated over the whole 245 seconds testing period, separately for each condition) increased with walking speed (F[2, 58] = 12.21, *p* < 0.001; η_p_^2^ = 0.30). No significant effects of surround contrast (F[3, 87] = 1.36, *p* = 0.264) or interaction (F[6, 174] = 1.16, *p* = 0.333) were found. *n* = 30 participants. (C) Reaction time increased with walking speed (F[2, 48] = 77.81, *p* < 0.001; η_p_^2^ = 0.76) and surround contrast (F[3, 72] = 6.48, *p* = 0.002; η_p_^2^ = 0.21). No significant interaction effect was found (F[6, 144] = 0.62, *p* = 0.652). (D) Behavioural detection-rate data (as shown in Fig 2A) were reorganised to reflect the absolute difference between background contrast level (black numbers on the x-axis) and target threshold. The surround contrast level is shown in blue. (E) Amplitudes of SSVEP around target presentation. The target-evoked SSVEP perturbation was larger for hit trials than for miss trials. No difference in amplitude was found across the four target locations. The shaded area marks the time window used for calculating the amplitude. (F) Detection rate for each target in each walking condition. The target in the right visual field had the highest detection rate. (G) A target preference index (between 0 and 1) was calculated to test whether participants had a disproportionally high detection rate in certain target locations. The shaded background represents the simulated preference index by assigning each detected target to a random location (1,000 simulations). Significant preference index was mainly found in the 100% contrast level condition (significant preference indices are accompanied by associated *p*-values). (H) The difference between the preference index calculated from real data and the average of simulated preference indices is shown. Vertical lines indicate ±1 standard error. SSVEP, steady-state visual evoked potential.
(TIF)

S5 Fig EEG power topography and walking step frequency. (A) Group average scalp topography of the raw power in 15 Hz (SSVEP), in the alpha band (8–12 Hz), and in the theta band (4–7 Hz) in each walking condition. Compared to SSVEP and alpha, theta power is notably high in the frontal area. *n* = 25 participants. (B) The power spectrum of walking speed data. Walking speed time-series data measured from both legs were added up and then analysed with a Fourier transform. *n* = 28 participants. The peak frequencies are 1 Hz and 1.75 Hz in the slow and normal walking conditions, respectively. Cyan lines mark the frequencies that showed power differences between walking conditions. EEG, electroencephalogram; SSVEP, steady-state visual evoked potential.
(TIF)

S1 Table Post hoc t test results of the interaction effects for SSVEP and detection rate. SSVEP, steady-state visual evoked potential.
(DOCX)

S1 Text Supplementary discussion. (DOCX)

## Abbreviations

AFz: anterior frontal midline
APA: American Psychological Association
EEG: electroencephalogram
EOG: electrooculogram
FDR: false discovery rate
MAD–median: median absolute deviation from the median
MNI: Montreal Neurological Institute
SSVEP: steady-state visual evoked potential

## References

[1] Keller GB, Bonhoeffer T, Hubener M. Sensorimotor mismatch signals in primary visual cortex of the behaving mouse. Neuron. 2012;74(5):809–15. 10.1016/j.neuron.2012.03.040 22681686

[2] Saleem AB, Ayaz A, Jeffery KJ, Harris KD, Carandini M. Integration of visual motion and locomotion in mouse visual cortex. Nat Neurosci. 2013;16(12):1864–9. 10.1038/nn.3567 24185423 PMC3926520

[3] Fu Y, Tucciarone JM, Espinosa JS, Sheng N, Darcy DP, Nicoll RA, et al A cortical circuit for gain control by behavioral state. Cell. 2014;156(6):1139–52. 10.1016/j.cell.2014.01.050 24630718 PMC4041382

[4] Lee AM, Hoy JL, Bonci A, Wilbrecht L, Stryker MP, Niell CM. Identification of a brainstem circuit regulating visual cortical state in parallel with locomotion. Neuron. 2014;83(2):455–66. 10.1016/j.neuron.2014.06.031 25033185 PMC4151326

[5] Niell CM, Stryker MP. Modulation of visual responses by behavioral state in mouse visual cortex. Neuron. 2010;65(4):472–9. 10.1016/j.neuron.2010.01.033 20188652 PMC3184003

[6] Polack P-O, Friedman J, Golshani P. Cellular mechanisms of brain state-dependent gain modulation in visual cortex. Nature neuroscience. 2013;16(9):1331–9. 10.1038/nn.3464 23872595 PMC3786578

[7] Ayaz A, Saleem AB, Scholvinck ML, Carandini M. Locomotion controls spatial integration in mouse visual cortex. Curr Biol. 2013;23(10):890–4. 10.1016/j.cub.2013.04.012 23664971 PMC3661981

[8] Dadarlat MC, Stryker MP. Locomotion Enhances Neural Encoding of Visual Stimuli in Mouse V1. J Neurosci. 2017;37(14):3764–75. 10.1523/JNEUROSCI.2728-16.2017 28264980 PMC5394894

[9] Vinck M, Batista-Brito R, Knoblich U, Cardin JA. Arousal and locomotion make distinct contributions to cortical activity patterns and visual encoding. Neuron. 2015;86(3):740–54. 10.1016/j.neuron.2015.03.028 25892300 PMC4425590

[10] Weir PT, Schnell B, Dickinson MH. Central complex neurons exhibit behaviorally gated responses to visual motion in Drosophila. J Neurophysiol. 2014;111(1):62–71. 10.1152/jn.00593.2013 24108792

[11] Chiappe ME, Seelig JD, Reiser MB, Jayaraman V. Walking modulates speed sensitivity in Drosophila motion vision. Curr Biol. 2010;20(16):1470–5. 10.1016/j.cub.2010.06.072 20655222 PMC4435946

[12] Maimon G, Straw AD, Dickinson MH. Active flight increases the gain of visual motion processing in Drosophila. Nat Neurosci. 2010;13(3):393–9. 10.1038/nn.2492 20154683

[13] Busse L, Cardin JA, Chiappe ME, Halassa MM, McGinley MJ, Yamashita T, et al Sensation during Active Behaviors. J Neurosci. 2017;37(45):10826–34. 10.1523/JNEUROSCI.1828-17.2017 29118211 PMC5678015

[14] Händel BF, Scholvinck ML. The brain during free movement—What can we learn from the animal model. Brain Res. 2019;1716:3–15. Epub 2017 Sep 8. 10.1016/j.brainres.2017.09.003 28893579

[15] McMorris T, Graydon J. The effect of incremental exercise on cognitive performance. International Journal of Sport Psychology. 2000;31(1):66–81.

[16] Conradi N, Abel C, Frisch S, Kell CA, Kaiser J, Schmidt-Kassow M. Actively but not passively synchronized motor activity amplifies predictive timing. Neuroimage. 2016;139:211–7. 10.1016/j.neuroimage.2016.06.033 27329809

[17] Schmidt-Kassow M, Heinemann LV, Abel C, Kaiser J. Auditory—motor synchronization facilitates attention allocation. Neuroimage. 2013;82:101–6. 10.1016/j.neuroimage.2013.05.111 23732882

[18] Labonté-LeMoyne É, Santhanam R, Léger P-M, Courtemanche F, Fredette M, Sénécal S. The delayed effect of treadmill desk usage on recall and attention. Computers in Human Behavior. 2015;46:1–5.

[19] Bullock T, Cecotti H, Giesbrecht B. Multiple stages of information processing are modulated during acute bouts of exercise. Neuroscience. 2015;307:138–50. 10.1016/j.neuroscience.2015.08.046 26318337

[20] Pelah A, Barbur J, Thurrell A, Hock HS. The coupling of vision with locomotion in cortical blindness. Vision Res. 2015;110(Pt B):286–94. 10.1016/j.visres.2014.04.015 24832646

[21] Pelah A, Barlow HB. Visual illusion from running. Nature. 1996;381(6580):283 10.1038/381283a0 8692265

[22] Thurrell A, Pelah A. Matching visual and nonvisual signals: evidence for a mechanism to discount optic flow during locomotion. Human Vision and Electronic Imaging X; 2005: International Society for Optics and Photonics.

[23] Thurrell A, Pelah A, Distler H. The influence of non-visual signals of walking on the perceived speed of optic flow. Perception. 1998;27:147–8. 10.1068/p270147 9709448

[24] Bullock T, Elliott JC, Serences JT, Giesbrecht B. Acute Exercise Modulates Feature-selective Responses in Human Cortex. J Cogn Neurosci. 2017;29(4):605–18. 10.1162/jocn_a_01082 27897672

[25] De Sanctis P, Butler JS, Malcolm BR, Foxe JJ. Recalibration of inhibitory control systems during walking-related dual-task interference: a mobile brain-body imaging (MOBI) study. Neuroimage. 2014;94:55–64. 10.1016/j.neuroimage.2014.03.016 24642283 PMC4209901

[26] Benjamin AV, Wailes-Newson K, Ma-Wyatt A, Baker DH, Wade AR. The Effect of Locomotion on Early Visual Contrast Processing in Humans. J Neurosci. 2018;38(12):3050–9. 10.1523/JNEUROSCI.1428-17.2017 29463642 PMC5864146

[27] Hollands MA, Marple-Horvat DE. Coordination of eye and leg movements during visually guided stepping. J Mot Behav. 2001;33(2):205–16. 10.1080/00222890109603151 11404215

[28] Matthis JS, Yates JL, Hayhoe MM. Gaze and the Control of Foot Placement When Walking in Natural Terrain. Curr Biol. 2018;28(8):1224–33 e5. 10.1016/j.cub.2018.03.008 29657116 PMC5937949

[29] Chen J, Valsecchi M, Gegenfurtner KR. Saccadic suppression measured by steady-state visual evoked potentials. J Neurophysiol. 2019;122(1):251–8. 10.1152/jn.00712.2018 30943105

[30] Lin YP, Wang Y, Wei CS, Jung TP. Assessing the quality of steady-state visual-evoked potentials for moving humans using a mobile electroencephalogram headset. Front Hum Neurosci. 2014;8:182 10.3389/fnhum.2014.00182 24744718 PMC3978365

[31] Gramann K, Gwin JT, Bigdely-Shamlo N, Ferris DP, Makeig S. Visual evoked responses during standing and walking. Front Hum Neurosci. 2010;4:202 10.3389/fnhum.2010.00202 21267424 PMC3024562

[32] Wascher E, Heppner H, Hoffmann S. Towards the measurement of event-related EEG activity in real-life working environments. Int J Psychophysiol. 2014;91(1):3–9. 10.1016/j.ijpsycho.2013.10.006 24144635

[33] Debener S, Minow F, Emkes R, Gandras K, de Vos M. How about taking a low-cost, small, and wireless EEG for a walk? Psychophysiology. 2012;49(11):1617–21. 10.1111/j.1469-8986.2012.01471.x 23013047

[34] De Vos M, Gandras K, Debener S. Towards a truly mobile auditory brain-computer interface: exploring the P300 to take away. Int J Psychophysiol. 2014;91(1):46–53. 10.1016/j.ijpsycho.2013.08.010 23994208

[35] Kranczioch C, Zich C, Schierholz I, Sterr A. Mobile EEG and its potential to promote the theory and application of imagery-based motor rehabilitation. Int J Psychophysiol. 2014;91(1):10–15. 10.1016/j.ijpsycho.2013.10.004 24144637

[36] Marigold DS, Patla AE. Visual information from the lower visual field is important for walking across multi-surface terrain. Exp Brain Res. 2008;188(1):23–31. 10.1007/s00221-008-1335-7 18322679

[37] Patla AE. How is human gait controlled by vision. Ecological Psychology. 1998;10(3–4):287–302.

[38] Warren WH Jr, Kay BA, Zosh WD, Duchon AP, Sahuc S. Optic flow is used to control human walking. Nature neuroscience. 2001;4(2):213 10.1038/84054 11175884

[39] Stoffregen TA, Schmuckler MA, Gibson EJ. Use of central and peripheral optical flow in stance and locomotion in young walkers. Perception. 1987;16(1):113–9. 10.1068/p160113 3671034

[40] Di Russo F, Pitzalis S, Aprile T, Spitoni G, Patria F, Stella A, et al Spatiotemporal analysis of the cortical sources of the steady-state visual evoked potential. Hum Brain Mapp. 2007;28(4):323–34. 10.1002/hbm.20276 16779799 PMC6871301

[41] Touyama H. A study on EEG quality in physical movements with Steady-State Visual Evoked Potentials. Conf Proc IEEE Eng Med Biol Soc. 2010;2010:4217–20. 10.1109/IEMBS.2010.5627375 21096897

[42] Vanegas MI, Blangero A, Kelly SP. Electrophysiological indices of surround suppression in humans. J Neurophysiol. 2014;113(4):1100–9. 10.1152/jn.00774.2014 25411464 PMC4329438

[43] Yogev-Seligmann G, Hausdorff JM, Giladi N. The role of executive function and attention in gait. Mov Disord. 2008;23(3):329–42. 10.1002/mds.21720 18058946 PMC2535903

[44] Lajoie Y, Teasdale N, Bard C, Fleury M. Attentional demands for static and dynamic equilibrium. Exp Brain Res. 1993;97(1):139–44. 10.1007/bf00228824 8131825

[45] Scanlon JEM, Townsend KA, Cormier DL, Kuziek JWP, Mathewson KE. Taking off the training wheels: Measuring auditory P3 during outdoor cycling using an active wet EEG system. Brain Res. 2019;1716:50–61. Epub 2017 Dec 14. 10.1016/j.brainres.2017.12.010 29248602

[46] Ehinger BV, Fischer P, Gert AL, Kaufhold L, Weber F, Pipa G, et al Kinesthetic and vestibular information modulate alpha activity during spatial navigation: a mobile EEG study. Front Hum Neurosci. 2014;8:71 10.3389/fnhum.2014.00071 24616681 PMC3934489

[47] Händel BF, Haarmeier T, Jensen O. Alpha oscillations correlate with the successful inhibition of unattended stimuli. J Cogn Neurosci. 2011;23(9):2494–502. 10.1162/jocn.2010.21557 20681750

[48] Klimesch W, Sauseng P, Hanslmayr S. EEG alpha oscillations: The inhibition-timing hypothesis. Brain Res Rev. 2007;53(1):63–88. 10.1016/j.brainresrev.2006.06.003 16887192

[49] Kelly SP, Lalor EC, Reilly RB, Foxe JJ. Increases in alpha oscillatory power reflect an active retinotopic mechanism for distracter suppression during sustained visuospatial attention. J Neurophysiol. 2006;95(6):3844–51. 10.1152/jn.01234.2005 16571739

[50] Sundberg KA, Mitchell JF, Reynolds JH. Spatial attention modulates center-surround interactions in macaque visual area v4. Neuron. 2009;61(6):952–63. 10.1016/j.neuron.2009.02.023 19324003 PMC3117898

[51] von Grünau MW, Pilgrim K, Zhou R. Velocity discrimination thresholds for flowfield motions with moving observers. Vision research. 2007;47(18):2453–64. 10.1016/j.visres.2007.06.008 17651779

[52] Petrov Y, Carandini M, McKee S. Two distinct mechanisms of suppression in human vision. J Neurosci. 2005;25(38):8704–7. 10.1523/JNEUROSCI.2871-05.2005 16177039 PMC1472809

[53] Kahana MJ, Sekuler R, Caplan JB, Kirschen M, Madsen JR. Human theta oscillations exhibit task dependence during virtual maze navigation. Nature. 1999;399(6738):781–4. 10.1038/21645 10391243

[54] Bush D, Bisby JA, Bird CM, Gollwitzer S, Rodionov R, Diehl B, et al Human hippocampal theta power indicates movement onset and distance travelled. Proc Natl Acad Sci U S A. 2017;114(46):12297–302. 10.1073/pnas.1708716114 29078334 PMC5699050

[55] Bulea TC, Kim J, Damiano DL, Stanley CJ, Park HS. Prefrontal, posterior parietal and sensorimotor network activity underlying speed control during walking. Front Hum Neurosci. 2015;9:247 10.3389/fnhum.2015.00247 26029077 PMC4429238

[56] Liang M, Starrett MJ, Ekstrom AD. Dissociation of frontal-midline delta-theta and posterior alpha oscillations: A mobile EEG study. Psychophysiology. 2018;55(9):e13090 10.1111/psyp.13090 29682758

[57] Lin CT, Chiu TC, Gramann K. EEG correlates of spatial orientation in the human retrosplenial complex. Neuroimage. 2015;120:123–32. 10.1016/j.neuroimage.2015.07.009 26163801

[58] Mitchell DJ, McNaughton N, Flanagan D, Kirk IJ. Frontal-midline theta from the perspective of hippocampal "theta". Prog Neurobiol. 2008;86(3):156–85. 10.1016/j.pneurobio.2008.09.005 18824212

[59] Xiao B, Wade AR. Measurements of long-range suppression in human opponent S-cone and achromatic luminance channels. Journal of vision. 2010;10(13):10 10.1167/10.13.10 21149312

[60] Haynes JD, Roth G, Stadler M, Heinze HJ. Neuromagnetic correlates of perceived contrast in primary visual cortex. Journal of Neurophysiology. 2003;89(5):2655–66. 12612045 10.1152/jn.00820.2002

[61] Xing J, Heeger DJ. Center-surround interactions in foveal and peripheral vision. Vision research. 2000;40(22):3065–72. 10.1016/s0042-6989(00)00152-8 10996610

[62] Snowden RJ, Hammett ST. The effects of surround contrast on contrast thresholds, perceived contrast and contrast discrimination. Vision research. 1998;38(13):1935–45. 10.1016/s0042-6989(97)00379-9 9797940

[63] Boto E, Holmes N, Leggett J, Roberts G, Shah V, Meyer SS, et al Moving magnetoencephalography towards real-world applications with a wearable system. Nature. 2018;555(7698):657–61. 10.1038/nature26147 29562238 PMC6063354

[64] Levitt H. Transformed up-down methods in psychoacoustics. J Acoust Soc Am. 1971;49(2B):467–77. 5541744

[65] Oostenveld R, Fries P, Maris E, Schoffelen JM. FieldTrip: Open source software for advanced analysis of MEG, EEG, and invasive electrophysiological data. Comput Intell Neurosci. 2011;2011:1 10.1155/2011/720971 21253357 PMC3021840

[66] Wilcox RR, Rousselet GA. A Guide to Robust Statistical Methods in Neuroscience. Curr Protoc Neurosci. 2018;82(1):8–42. 29357109 10.1002/cpns.41

[67] Oostenveld R, Stegeman DF, Praamstra P, van Oosterom A. Brain symmetry and topographic analysis of lateralized event-related potentials. Clin Neurophysiol. 2003;114(7):1194–202. 12842715 10.1016/s1388-2457(03)00059-2

[68] Holmes CJ, Hoge R, Collins L, Woods R, Toga AW, Evans AC. Enhancement of MR images using registration for signal averaging. J Comput Assist Tomogr. 1998;22(2):324–33. 10.1097/00004728-199803000-00032 9530404

[69] Yekutieli D, Benjamini Y. Resampling-based false discovery rate controlling multiple test procedures for correlated test statistics. Journal of Statistical Planning and Inference. 1999;82(1–2):171–96.

[70] Keren AS, Yuval-Greenberg S, Deouell LY. Saccadic spike potentials in gamma-band EEG: characterization, detection and suppression. Neuroimage. 2010;49(3):2248–63. 10.1016/j.neuroimage.2009.10.057 19874901

