# Supplementary figures and images for: Walking enhances peripheral visual processing in humans
Source: PLoS Biol. 2019 Oct 11;17(10):e3000511. doi: 10.1371/journal.pbio.3000511 (PMC6808500; doi:10.1371/journal.pbio.3000511)

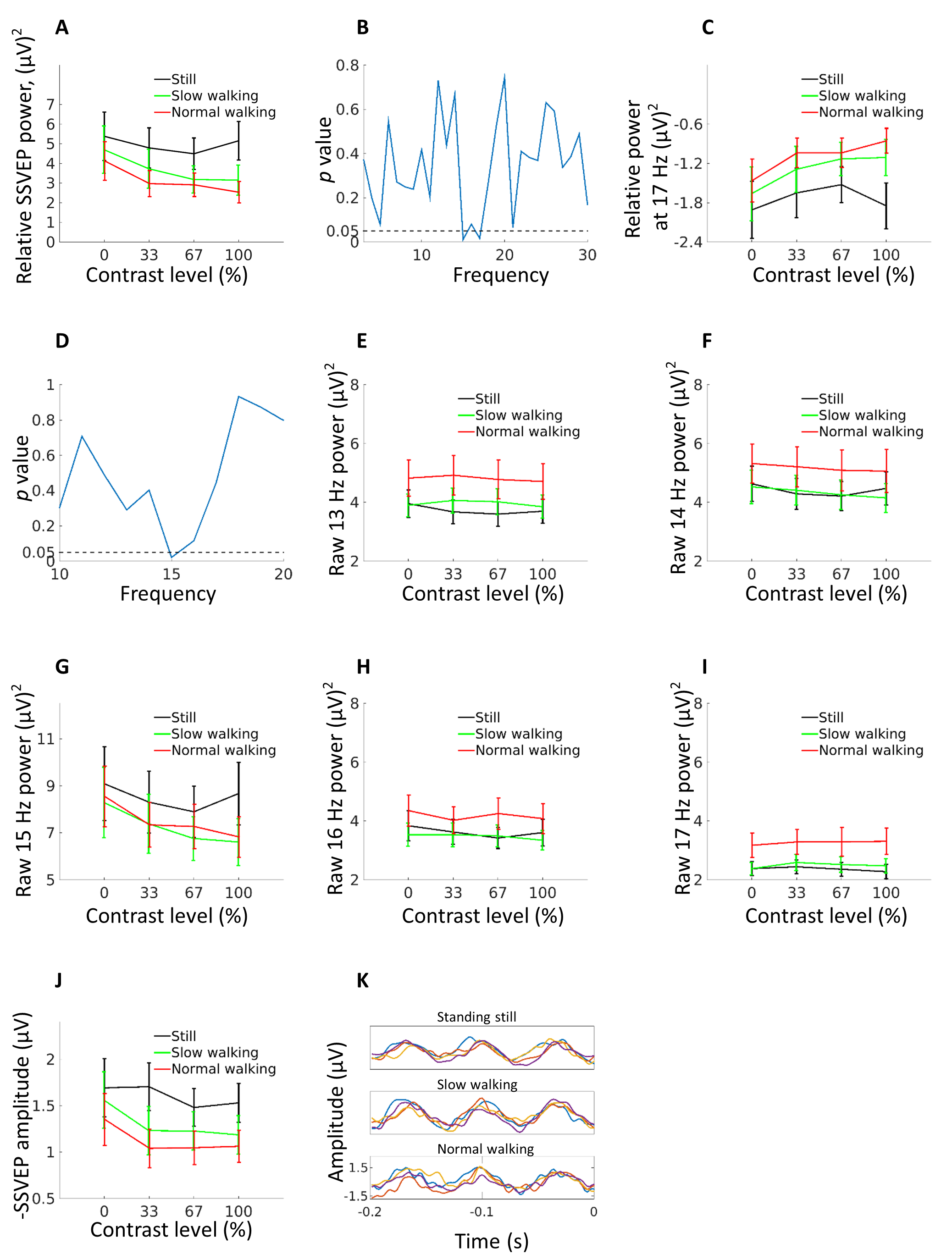

Supplement: S1 Fig — (A) Referenced SSVEP power without controlling for eye movements also showed a significant interaction between walking condition and surround contrast. (B) p-Values for the interaction effect between walking condition and contrast level for signals from 3 to 30 Hz (step size 1 Hz) processed in the same way as for the 15 Hz relative SSVEP signal. All frequencies had p-values above 0.05 except the signals of 15 and 17 Hz, the latter of which took contribution from 15 Hz signal (signals from each frequency were referenced to the mean of four nearby frequencies). (C) The relative power at 17 Hz (referenced to the average power of 15, 16, 18, and 19 Hz) also showed a significant interaction effect between walking condition and contrast level. This effect is likely driven by the SSVEP signal at 15 Hz. Note the negative sign of the relative power and the positive influence of surround contrast. (D) p-Values for the interaction effect between walking condition and contrast level for the raw power from 10 to 20 Hz (step size 1 Hz). All frequencies had p-values above 0.05, except the signal of 15 Hz. (E–I) Raw power shown in each walking condition and surround contrast combination for 13–17 Hz. (J) Amplitudes for target-evoked SSVEP perturbation (averaged between hit and miss trials; sign reversed; amplitude taken as the lowest amplitude point in a post-target time window of [0.2, 1] second). Note that another four participants were excluded (21 participants remained) because no hit or miss trials could be found in at least one condition for them. (K) EEG signal aligned to the onset of the last central contrast before the onset of the behavioural target, i.e., time 0 is the onset time of a central contrast. It is clear that the phase of EEG signal is aligned between all levels of surround contrast in all walking conditions. Border contrast elicited response would lead to a 180° phase difference between the 0% contrast and the 100% contrast condition. Blue colour: 0% contrast; [file pbio.3000511.s001.tif]

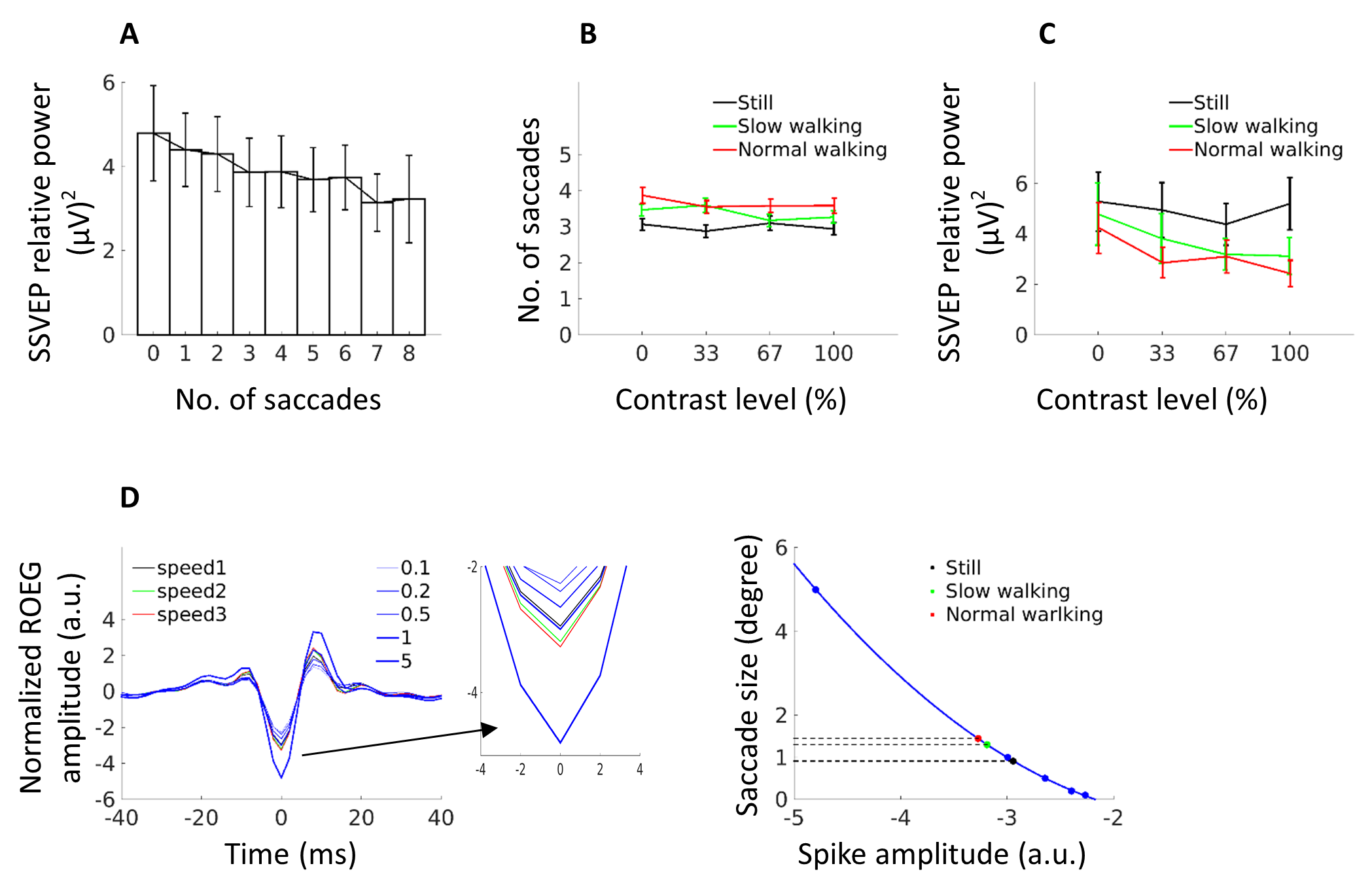

Supplement: S2 Fig — (A) Trials were grouped based on the number of saccades detected within each trial (97.4% of all trials from 25 participants included. Trials showing more than eight saccades were not considered). SSVEP relative power decreased with increased number of saccades per trial. (B) Average number of saccades in each trial for each walking condition/contrast level combination. Only main effects of walking condition (F[2, 48] = 22.95, p < 0.001; ηp2 = 0.49) and contrast level (F[3, 72] = 3.78, p = 0.018; ηp2 = 0.14) were significant. n = 25 participants. (C) SSVEP in walking condition/contrast level combination with the potential influence from saccades controlled. (D) (Left) The saccadic spike potential (the U-shaped waveform) averaged across 25 participants for controlled saccades to visually presented targets (saccade amplitude: 0.1°, 0.2°, 0.5°, 1.0°, and 5.0°) and for saccades in each walking condition. The amplitude value at time 0 was used for the fitting shown on the right. (Right) The group average saccade size in each walking condition was estimated based on the amplitude at time point 0 of the controlled saccades with known sizes. The blue dots represent the controlled saccades, and the blue line is the fitting curve. Vertical lines indicate ±1 standard error. SSVEP, steady-state visual evoked potential. (TIF) [file pbio.3000511.s002.tif]

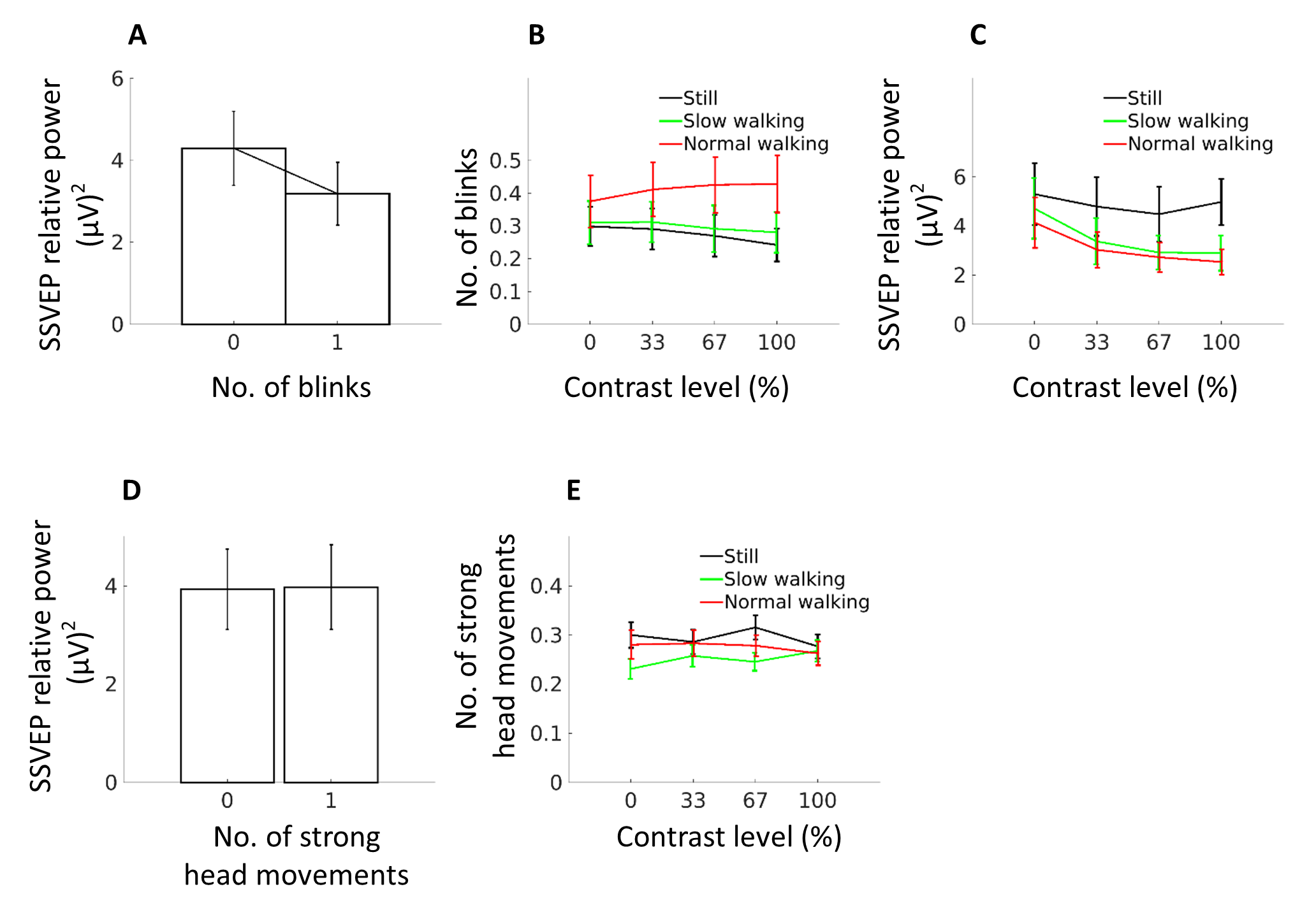

Supplement: S3 Fig — (A) Trials were grouped based on the number of blinks detected within each trial (95.2% of all trials from 25 participants). SSVEP relative power was lower in no-blink trials than in trials with one blink. (B) Average number of blinks in each trial for each walking condition/contrast level combination. Only the main effect of walking condition (F[2, 48] = 4.21, p = 0.038; ηp2 = 0.15) was significant. n = 25 participants. (C) SSVEP in walking condition/contrast level combination with the influence from blinks controlled. This is the same figure as Fig 2A (left) in the main text. (D) Trials were grouped based on the number of strong head movements detected within each trial (all trials from 25 participants). The SSVEP relative power did not change with number of strong head movements (t[24] = −0.45, p = 0.659). (E) There was no significant interaction effect for strong head movements between walking condition and surround contrast (F[6, 144] = 0.95, p = 0.449). The main effects of walking condition (F[2, 48] = 1.68, p = 0.199) and contrast level (F[3, 72] = 0.37, p = 0.766) were also not significant. Vertical lines indicate ±1 standard error. SSVEP, steady-state visual evoked potential. (TIF) [file pbio.3000511.s003.tif]

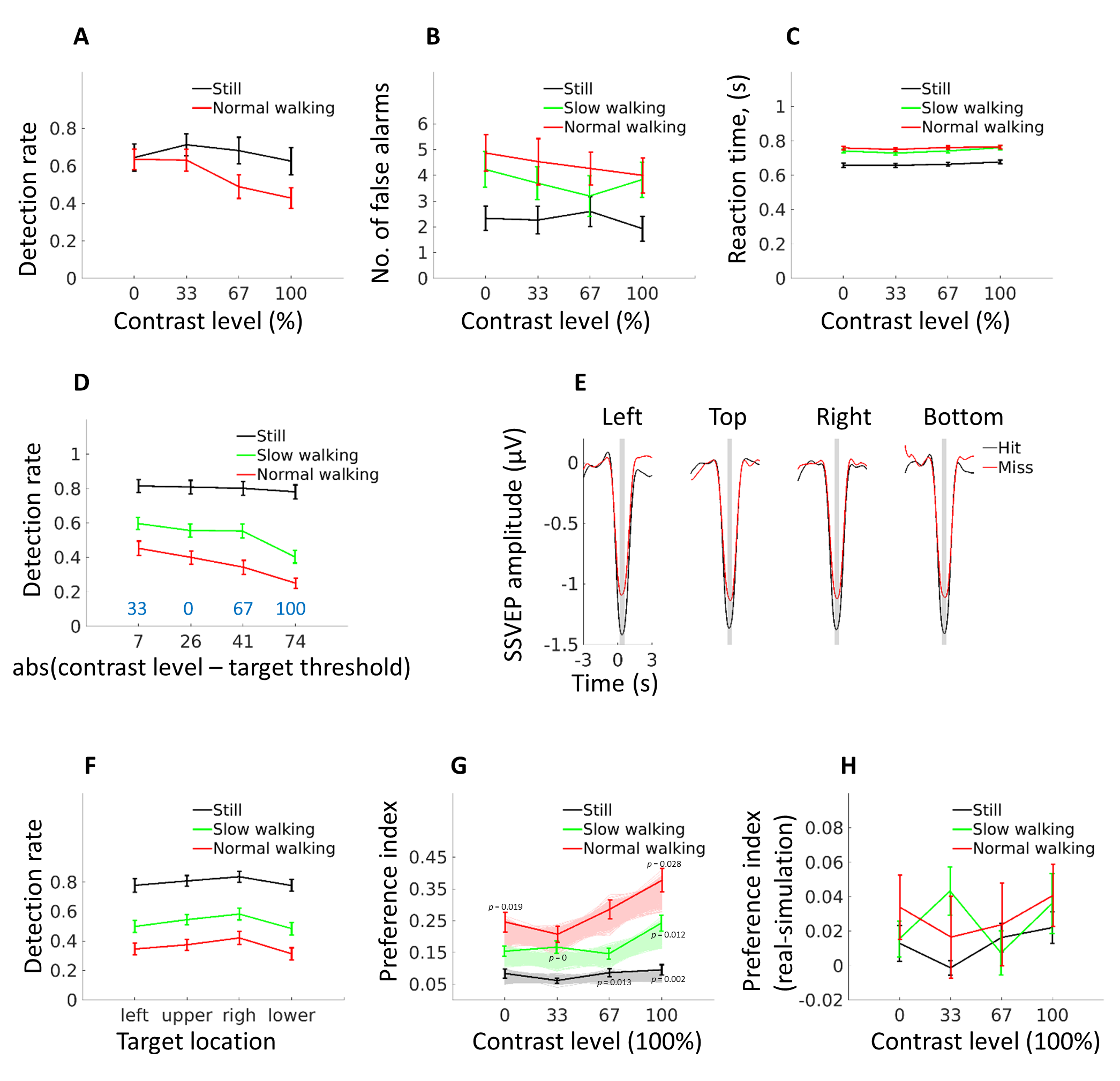

Supplement: S4 Fig — (A) When the detection rate was forced to be comparable at 0% surround contrast (t[20] = 0.15, p = 0.882), detectable differences at 67% (t[20] = 2.54, p = 0.020; dz = 0.55) and 100% (t[20] = 3.57, p = 0.002; dz = 0.78) surround contrast can still be found between the still and normal walking conditions. A significant interaction between surround contrast and walking speed was also present (F[3, 60] = 3.19, p = 0.040; ηp2 = 0.14). n = 21 participants. (B) Number of false alarms (calculated over the whole 245 seconds testing period, separately for each condition) increased with walking speed (F[2, 58] = 12.21, p < 0.001; ηp2 = 0.30). No significant effects of surround contrast (F[3, 87] = 1.36, p = 0.264) or interaction (F[6, 174] = 1.16, p = 0.333) were found. n = 30 participants. (C) Reaction time increased with walking speed (F[2, 48] = 77.81, p < 0.001; ηp2 = 0.76) and surround contrast (F[3, 72] = 6.48, p = 0.002; ηp2 = 0.21). No significant interaction effect was found (F[6, 144] = 0.62, p = 0.652). (D) Behavioural detection-rate data (as shown in Fig 2A) were reorganised to reflect the absolute difference between background contrast level (black numbers on the x-axis) and target threshold. The surround contrast level is shown in blue. (E) Amplitudes of SSVEP around target presentation. The target-evoked SSVEP perturbation was larger for hit trials than for miss trials. No difference in amplitude was found across the four target locations. The shaded area marks the time window used for calculating the amplitude. (F) Detection rate for each target in each walking condition. The target in the right visual field had the highest detection rate. (G) A target preference index (between 0 and 1) was calculated to test whether participants had a disproportionally high detection rate in certain target locations. The shaded background represents the simulated preference index by assigning each detected target to a random location (1,000 simulations). Significant preferenc [file pbio.3000511.s004.tif]

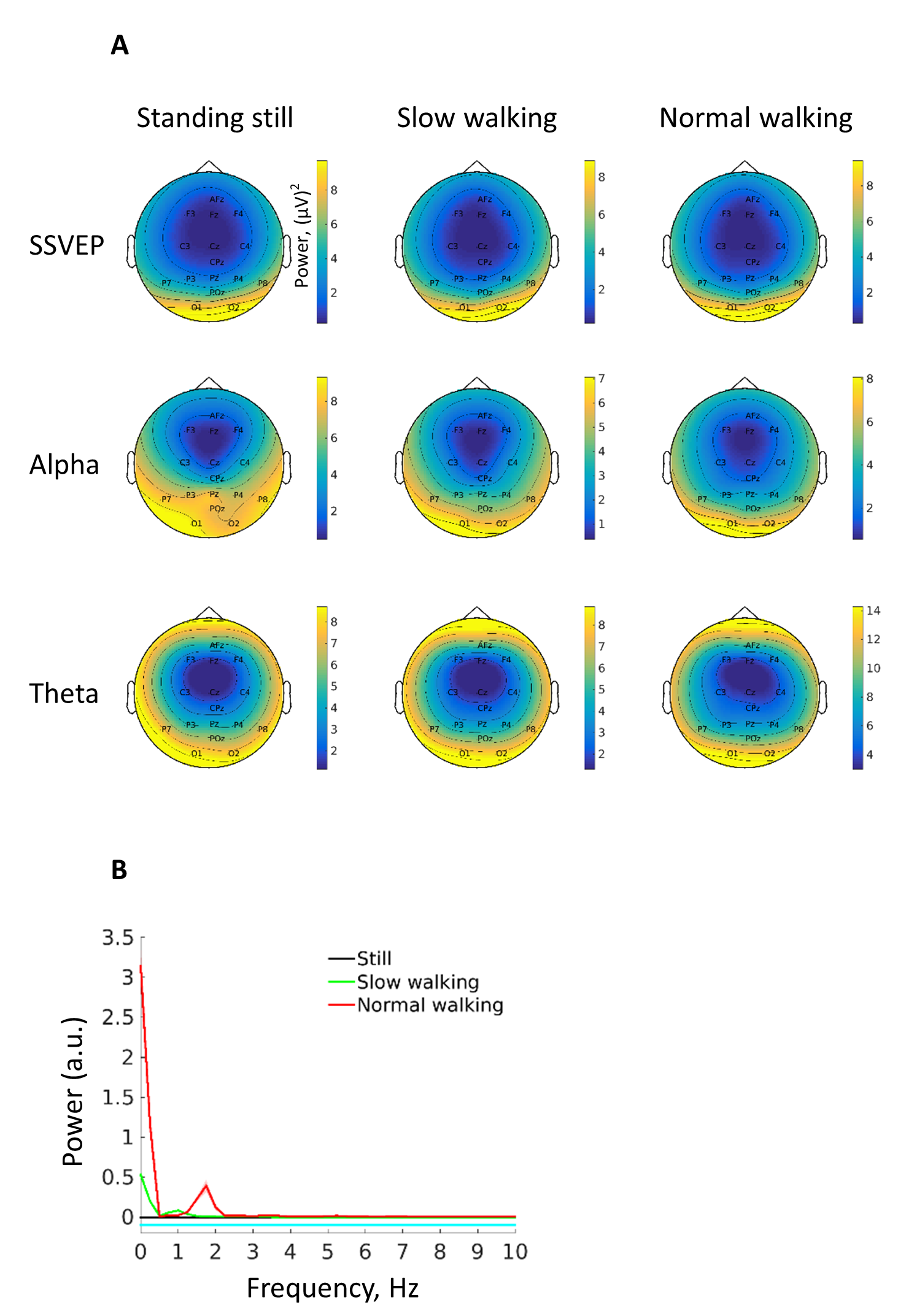

Supplement: S5 Fig — (A) Group average scalp topography of the raw power in 15 Hz (SSVEP), in the alpha band (8–12 Hz), and in the theta band (4–7 Hz) in each walking condition. Compared to SSVEP and alpha, theta power is notably high in the frontal area. n = 25 participants. (B) The power spectrum of walking speed data. Walking speed time-series data measured from both legs were added up and then analysed with a Fourier transform. n = 28 participants. The peak frequencies are 1 Hz and 1.75 Hz in the slow and normal walking conditions, respectively. Cyan lines mark the frequencies that showed power differences between walking conditions. EEG, electroencephalogram; SSVEP, steady-state visual evoked potential. (TIF) [file pbio.3000511.s005.tif]
