# Supplementary material for: Walking enhances peripheral visual processing in humans
Source: PLoS Biol. 2019 Oct 11;17(10):e3000511. doi: 10.1371/journal.pbio.3000511 (PMC6808500; doi:10.1371/journal.pbio.3000511)
Supplement: S1 Table — SSVEP, steady-state visual evoked potential. (DOCX) [file pbio.3000511.s006.docx]

| Post-hoc t-tests for SSVEP interaction (walking condition x surround contrast).  SSVEP power was referenced to side bands and controlled for blink rates.  (degree of freedom = 24; t values are reported with FDR-adjusted *p* values in brackets) | | | | | | |
| --- | --- | --- | --- | --- | --- | --- |
|  | 0 vs. 33 | 0 vs. 67 | 0 vs. 100 | 33 vs. 67 | 33 vs. 100 | 67 vs. 100 |
| Standing still | 1.32  (0.292) | 2.08  (0.084) | 0.64  (0.624) | 1.02  (0.411) | -0.49  (0.670) | -1.30  (0.291) |
| Slow walking | **3.64**  **(0.012)** | **2.85**  **(0.039)** | **2.93**  **(0.037)** | 1.29  (0.292) | 1.48  (0.247) | 0.12  (0.905) |
| Normal walking | **2.47**  **(0.047)** | **2.72**  **(0.040)** | **2.48**  **(0.047)** | 0.84  (0.502) | 1.36  (0.287) | 0.74  (0.563) |
| Post-hoc t-tests for SSVEP interaction (walking condition x surround contrast).  The raw SSVEP power was used here.  (degree of freedom = 24; t values are reported with FDR-adjusted *p* values in brackets) | | | | | | |
|  | 0 vs. 33 | 0 vs. 67 | 0 vs. 100 | 33 vs. 67 | 33 vs. 100 | 67 vs. 100 |
| Standing still | 1.56 (0.271) | 2.49 (0.081) | 0.64 (0.631) | 1.29 (0.350) | -0.80 (0.573) | -1.93 (0.189) |
| Slow walking | **2.99 (0.043)** | 2.55 (0.077) | 2.65 (0.071) | 1.40 (0.341) | 1.62 (0.254) | 0.05 (0.974) |
| Normal walking | 2.74 (0.068) | 2.70 (0.069) | 2.47 (0.081) | 0.52 (0.695) | 1.08 (0.427) | 0.92 (0.503) |
| Post-hoc t-tests for detection rate interaction (walking condition x surround contrast)  (degree of freedom = 29; t values are reported with FDR-adjusted *p* values in brackets) | | | | | | |
|  | 0 vs. 33 | 0 vs. 67 | 0 vs. 100 | 33 vs. 67 | 33 vs. 100 | 67 vs. 100 |
| Standing still | -0.44 (0.685) | 0.47 (0.675) | **2.14 (0.046)** | 0.94 (0.380) | **2.49 (0.022)** | 1.50 (0.160) |
| Slow walking | **-2.75 (0.012)** | 0.18 (0.875) | **8.60 (<0.001)** | **2.87 (0.009)** | **10.93 (<0.001)** | **6.46 (<0.001)** |
| Normal walking | **-2.24 (0.038)** | **2.64 (0.016)** | **7.70 (<0.001)** | **6.01 (<0.001)** | **8.73 (<0.001)** | **4.78 (<0.001)** |
