# Supplementary material for: Walking enhances peripheral visual processing in humans
Source: PLoS Biol. 2019 Oct 11;17(10):e3000511. doi: 10.1371/journal.pbio.3000511 (PMC6808500; doi:10.1371/journal.pbio.3000511)
Supplement: S1 Text — (DOCX) [file pbio.3000511.s007.docx]

**Supplementary Discussion**

SSVEP interaction effect is a robust visual response independent from SNR

EEG recordings during walking can be greatly influenced by non-sensory brain activity, such as walking related outside artefacts (e.g. cable movement or impedance changes due to sweating), inside artefacts (like heartbeat and muscle activity mainly from eyes or neck), and brain activity related to the motor aspects of the body movement (e.g. beta activity over motor cortex). In Fig 2A (left) of the main text, we showed that there was a significant interaction effect on blink controlled referenced SSVEP response between walking condition and surround contrast. The interaction was also significant with the referenced SSVEP without controlling for blinks (F(6,144) = 3.51, *p* = 0.012; S1A Fig). Moreover, the interaction effect between walking condition and surround contrast was frequency specific for 15 Hz, as revealed by repeating the ANOVA from 3 to 30 Hz (step size 1, S1B Fig). This 15 Hz signal (SSVEP) was directly induced by the visual stimulus and known to originate from early visual cortex [1]. Please note that the opposite influence at 17 Hz as revealed from the ANOVA was due to referencing the power to neighboring frequencies including 15 Hz (S1C Fig), i.e. the effect at 17 Hz takes contribution from the effect at 15 Hz and therefore still represents the effect from 15 Hz. The reported SSVEP interaction effect is therefore easily distinguishable from walking related artefacts. One might argue that different walking conditions can lead to different signal to noise ratios (SNR), which consequently, can affect how much visually induced activity can be picked up. Recent studies dissent on the specific effect of different types of movements on the EEG signal [2, 3], but without doubt, body movements can alter the signal to noise ratio. Note that while there is an indication of a broad-band power increase during walking (Fig 1C), SSVEP power was referenced to the mean power of nearby frequencies, thereby considering a changed signal to noise ratio. Moreover, we compared power only between surround contrast conditions and not between walking conditions, which excludes any low level influences of walking on our results. To further rule out the possibility that the interaction effect of SSVEP takes contribution from noises at sidebands, the same interaction analysis was performed with raw power at frequencies around 15 Hz. As can be found in S1D-I Fig, a significant interaction effect can only be found at 15 Hz.

There is a decrease in the SSVEP amplitude following the onset of the target for the detection task (S4E Fig). Since the amplitude of SSVEP perturbation was significantly different between target hit and target miss trials, a 2 (target hit vs. target miss) by 3 (walking condition) by 4 (contrast level) ANOVA was performed on the amplitude of SSVEP perturbation. A significant interaction was found between walking condition and surround contrast (F(6,120) = 2.58, *p* = 0.046). Whereas no modulation effect was found for the surround contrast in the still condition (F(3,60) = 1.89, *p* = 0.165), significant suppression effect was found during both slow walking (F(3,60) = 4.21, *p* = 0.024) and normal walking (F(3,60) = 4.00, p = 0.038) (S1J Fig).

Control analyses to exclude movement related artefacts

Since the focus of the current study was on visual perception during natural walking, large body movements but also saccades and other eye related movements were not controlled. It is therefore highly important to exclude the possibility that movement related artefacts explain our results.

Sensory brain activity such as the SSVEP can be modulated by walking induced differences in visual input. Such changed visual input might be due to 1) different eye movements, 2) movements of the head-mounted display, or 3) optic flow from outside the head mounted display. The analyses described below covered these 3 possibilities and showed that these factors cannot explain our main finding.

Saccade rates had an influence on the SSVEP power. In the 2-second-long trials selected for the SSVEP analysis, increased number of saccades led to decreased SSVEP power (within-subjects one-way ANOVA: F(8,192) = 4.16, *p* = 0.003; η_p_^2^ = 0.15; S2A Fig). However, there was no significant interaction effect for saccade numbers between walking condition and contrast level (F(6,144) = 1.92, *p* = 0.109; S2B Fig). Saccade numbers even seem to decrease with increased contrast levels while walking, which should lead to increased SSVEP power for higher contrast levels. Therefore, saccade rate cannot explain the SSVEP interaction effect. As a final check, the influence of saccade rate on the SSVEP response was controlled. This was done by first taking the average of the trials with different number of saccades separately and then averaging those averages. Only trials with the number of saccades that could be found in all walking condition/surround contrast combinations were included. The ANOVA analysis was repeated with the saccade controlled SSVEP and again a significant interaction effect was found between walking condition and surround contrast (F(6,144) = 3.79, *p* = 0.017) (S2C Fig). The group average saccade size was estimated to be 0.9^o^ (still condition), 1.3^o^ (slow walking) and 1.4^o^ (normal walking) (S2D Fig). Note that the diameter of the central grating is 6.1^o^, suggesting that the central grating always falls onto the fovea even if saccades are executed.

Blinks, just like saccades, were associated with a decrease in SSVEP power (within-subjects t-test: t(24) = 5.00, *p* < 0.001; d_z_ = 1.00; S3A Fig). However, there was no significant interaction effect between walking condition and contrast level for blink numbers (F(6,144) = 1.62, *p* = 0.172; S3B Fig). Therefore, blink rate cannot explain the SSVEP interaction effect. To fully control the influence of blinks on SSVEP amplitude, trials with 0 or 1 blink detected were averaged separately before the two averages were averaged. Still, a significant interaction between walking condition and surround contrast can be found in this case (F(6,144) = 3.03, *p* = 0.021) (S3C Fig, which was already shown as Fig 2A (left) in the main text). The above reported analysis clearly indicates that eye movements do not mediate the effect of walking on surround suppression.

Besides eye movements, a movement of the visual display could also introduce changed sensory input. In our study, we used a head-mounted display to present the visual stimulation. The specific equipment (Glyph Founder’s edition) was designed such that all parts are relatively close to the head and lightweight in comparison to other head-mounted displays (e.g. Oculus rift, HTCvive). This made movement of the device during walking less likely. In fact, no movement could be visually observed during walking. Additionally, since our stimuli were vertical stripes, any up and down movement would only lead to minimal changes in the visual input and only at the boarders of the display. However, jerky head movements to either side could introduce horizontal movements. Analysis of strong head movements showed that they were not associated with SSVEP power changes and that no significant interaction effects between walking condition and surround contrast was present for the rate of head movements (S3D and S3E Fig).

Behavioural target related response

It was demonstrated that the behavioural detection rate was modulated by walking condition and contrast level in a similar fashion as the SSVEP response. However, as can be observed in Fig 2A, the shape of modulation was not the same. We want to point out that behavioural detection rate and SSVEP response are two separate processes that may be controlled by different mechanisms. One difference arose from the fact that the target was also a contrasted grating. Therefore, target detection rate was related to the absolute difference between (surround) contrast level and target threshold (i.e. the contrast difference between target and central grating). The mean target threshold was 26.3% (SD: 5.0%). When detection rate was organized to reflect the absolute difference between (surround) contrast level and target threshold, a pattern of monotonic decrease of detection rate can be observed (S4D Fig, cf. Fig 2A). 7% absolute contrast difference gave higher detection rate than 26% (t(29) = 3.16, *p* = 0.004; d_z_ = 0.58), 41% (t(29) = 6.24, *p* < 0.001; d_z_ = 1.14) and 74% (t(29) = 13.31, *p* < 0.001; d_z_ = 2.43). 26% absolute contrast difference gave higher detection rate than 41% (t(29) = 2.05, *p* = 0.0498; d_z_ = 0.37) and 74% (t(29) = 10.30, *p* < 0.001; d_z_ = 1.88). 41% absolute contrast difference gave higher detection rate than 74% (t(29) = 7.35, *p* < 0.001; d_z_ = 1.34). Furthermore, at the 33% surround contrast level, a significant negative correlation across participant was found between target detection rate and the absolute contrast difference (r = -0.41, *p* = 0.026).

As is described in the methods section, participants walked in a dimly lit sports hall where the floor was of uniform brown wood with infrequent colored lines marking different sports courses. Participants were able to move freely because the lower rim of the visual field was unobstructed. Anatomical difference of the face led to a variable distance of the head mounted display to the eyes, and consequently to a different amount of visual input. A rough calculation suggests that an area of 33 to 48 degrees of the lower visual field and between 21 and 36 degrees of the upper visual field was visible. This means that there was considerable input of optic flow during walking that was modulated by walking speed, although the input was of low contrast. Such moving visual input could attract gaze or attention. Independent of whether gaze or attention is shifted, both shifts should lead to enhanced processing of targets appearing at or close to the upper or lower visual field. We therefore checked if targets would differ in their neurophysiological responses depending on location. Target onset caused a perturbation of the SSVEP signal, which we call the target evoked SSVEP perturbation (S4E Fig). Comparing the amplitude of the target evoked SSVEP perturbation, a 2 (target hit vs. target miss) by 4 (target locations) ANOVA showed only a significant effect of target hit/miss (F(1,24) = 20.65, *p* < 0.001; η_p_^2^ = 0.46). Neither the main effect of target location (F(3,72) = 0.35, *p* = 0.733) nor the interaction effect (F(3,72) = 0.60, *p* = 0.609) was significant. For the behavioural performance, we conducted a detailed analysis on the influence of target location on detection rate. The detection rate for each target in different walking conditions was computed (S4A Fig). A 3 (walking condition) by 4 (target location) ANOVA revealed significant main effects of walking condition (F(2,58) = 102.42, *p* < 0.001; η_p_^2^ = 0.78) and target location (F(3,87) = 7.27, *p* < 0.001; η_p_^2^ = 0.20). There was no interaction effect ((F(6,174) = 0.59, *p* = 0.695). For target locations, post-hoc t-tests showed that the detection rate for the right target was higher than the left (t(29) = 3.02, *p* = 0.005; d_z_ = 0.55) and the bottom target ( t(29) = 3.99, *p* < 0.001; d_z_ = 0.73) and that the top target was higher than the lower target (t(29) = 2.58, *p* = 0.015; d_z_ = 0.47). The average detection advantage for the right and top targets may reflect a general attentional bias rather than a group-level shift of fixation point. Importantly, it was not the bottom target that had a higher detection rate. Further, a target preference index was calculated for each participant in each walking condition and contrast level combination based on the detection rate of each target, reasoning that different participants may have preferences for different locations. The preference index ranges between 0 (when detection rates for all the targets were the same) and 1 (when detections were made only for one of the targets). The preference index was statistically tested against simulated data assuming no target preference was present and was found to be significant in some conditions (S4B Fig). However, a 3 (walking condition) by 4 (contrast level) ANOVA analysis on the difference between real preference index and the simulated preference index showed no significant main effects (walking condition: F(2,56) = 1.07, *p* = 0.344; contrast level: F(3,84) = 0.83, *p* = 0.473) or interaction effect (F(6,168) = 0.78, *p* = 0.536). As can be seen in S4C Fig, the overall preference index difference between the real data and the simulated data was very small (around 0.02), considering that the preference index ranges between 0 and 1. Therefore, target location preference, if there is any, cannot explain our main results.

**References**

1. Di Russo F, Pitzalis S, Aprile T, Spitoni G, Patria F, Stella A, et al. Spatiotemporal analysis of the cortical sources of the steady-state visual evoked potential. Hum Brain Mapp. 2007;28(4):323-34. doi: 10.1002/hbm.20276. PubMed PMID: 16779799.

2. Oliveira AS, Schlink BR, Hairston WD, Konig P, Ferris DP. Proposing Metrics for Benchmarking Novel EEG Technologies Towards Real-World Measurements. Front Hum Neurosci. 2016;10:188. doi: 10.3389/fnhum.2016.00188. PubMed PMID: 27242467; PubMed Central PMCID: PMCPMC4861738.

3. Nathan K, Contreras-Vidal JL. Negligible Motion Artifacts in Scalp Electroencephalography (EEG) During Treadmill Walking. Front Hum Neurosci. 2015;9:708. doi: 10.3389/fnhum.2015.00708. PubMed PMID: 26793089; PubMed Central PMCID: PMCPMC4710850.
